# TERT drives liver tumorigenesis beyond telomere elongation

**DOI:** 10.26508/lsa.202603660

**Published:** 2026-07-02

**Authors:** Laura Braud, Julien Vernerey, Arnaud Guille, Pierre Cordier, Clémence Ginet, Tom Egger, Manuel Bernabe, Dmitri Churikov, Quentin Da Costa, Aïda Meghraoui, Chantal Desdouets, Li Gu, François Bertucci, Christophe Lachaud, Vincent Géli

**Affiliations:** 1 CRCM, Inserm, CNRS, Institut Paoli-Calmettes, Aix-Marseille University Marseille, Équipe labellisée par la Ligue Nationale Contre le Cancer, Marseille, France; 2 Predictive Oncology Laboratory, Marseille Cancer Research Centre (CRCM), Inserm U1068, CNRS UMR7258, Institut Paoli-Calmettes, Aix-Marseille University, Marseille, France; 3 Centre de recherche des Cordeliers, Sorbonne université, Inserm, Université Paris Cité, Équipe labellisée par la Ligue Nationale Contre le Cancer, Paris, France; 4 AMKbiotech, BIOPARC, Biot, France; 5 Quantimagin, Montpellier, France; 6 Department of Laboratory Medicine and Clinical Laboratory Medicine Research Center, West China Hospital, Sichuan University, Chengdu, China; 7 CRCM, Inserm, CNRS, Institut Paoli-Calmettes, Aix-Marseille University, DNA Interstrand Crosslink Lesions and Blood Disorder Team, Marseille, France; 8 Institute for Research on Cancer and Aging of Nice (IRCAN), Université Côte d’Azur, INSERM, CNRS, Nice, France

## Abstract

We generated two mouse models, p21^+/Tert^ and p21^+/TertCi^, expressing either telomerase reverse transcriptase (Tert) or a catalytically inactive variant under the control of the p21 promoter. By 18–20 mo of age, ∼15% of mice from both genotypes developed liver tumors with histopathological features resembling human hepatocellular carcinoma (HCC). Whole-exome sequencing identified activating Ctnnb1 mutations and recurrent PP1 subunit alterations in p21^+/Tert^ tumors, whereas p21^+/TertCi^ tumors harbored activating Hras^Gln61Lys^ mutations associated with elevated C > A transversions. Both models exhibited chromosomal aberrations commonly observed in human HCC. Transcriptomic analyses revealed that β-catenin–activated tumors recapitulated gene expression signatures of human HCC, whereas MAPK-mutated tumors showed profiles consistent with MAPK/ERK pathway activation. All HCCs suppressed the gluconeogenic genes *Fbp1* and *AldoB*, but diverged into two distinct groups based on their glycolytic and *NRF2* target gene expression profiles. Spatial profiling further revealed reduced HNF4α-positive hepatocytes across tumors, independent of HNF4α transcription, and markedly diminished immune cell infiltration, particularly in β-catenin–activated tumors. Collectively, these findings uncover telomere-independent functions of Tert and identify molecular and metabolic features with potential relevance for predicting immunotherapy response.

## Abstract (precis)

Mice expressing Tert or its inactive variant under the 21 promoter develop HCC with distinct mutational profiles, prompting a reassessment of Tert’s role in hepatocellular carcinoma generation.

## Introduction

Hepatocellular carcinoma (HCC) is the third leading cause of cancer-related mortality worldwide. It arises in the setting of chronic liver diseases, such as chronic hepatitis B or C infection, cirrhosis, and metabolic syndrome, which are frequently accompanied by persistent inflammation (Zucman-Rossi et al, 2015; Nault et al, 2019). Within this pathological environment, the accumulation of driver mutations leads to the disruption of key signaling pathways, ultimately driving hepatocarcinogenesis (Schulze et al, 2015). Among the most frequently deregulated are the Wnt/β-catenin and p53 pathways (Fujimoto et al, 2015; Rebouissou et al, 2016). In addition, multiple other pathways contribute to HCC development, including recurrent amplifications of MYC and cyclin-dependent kinase genes, and mutations that activate the Sonic Hedgehog, RAS/MAPK, PI3K-Akt, and oxidative stress response pathways (Chen et al, 2010; Delire & Stärkel 2015; Zhang et al, 2016).

Frequent mutations in HCC occur early in carcinogenesis within the promoter region of the TERT gene, which encodes the catalytic subunit of telomerase (Nault et al, 2013, 2014). Various genetic and epigenetic mechanisms, including TERT promoter mutations, hepatitis B virus integration, chromosomal rearrangements, and promoter methylation changes, lead to aberrant up-regulation of TERT in the majority of HCC cases (Totoki et al, 2014). Traditionally, telomerase reactivation in HCC is considered to be a compensatory response to critical telomere shortening, preventing senescence and apoptosis, especially in cells harboring p53 mutations (Farazi et al, 2006). However, emerging evidence indicates that TERT also performs noncanonical functions beyond telomere elongation that may promote tumorigenesis (Ségal-Bendirdjian & Géli, 2019). Depending on cellular context, mouse Tert has been implicated in the regulation of major oncogenic pathways, including WNT/β-catenin, NF-κB, and MYC (Park et al, 2009; Ghosh et al, 2012; Koh et al, 2015). Recent studies in murine models of chronic liver inflammation have shown that Tert can enhance NF-κB promoter activity and drive liver tumor formation, particularly in the absence of functional p53 (Mishima et al, 2024). Despite these findings, the noncanonical roles of TERT in HCC remain poorly understood.

We developed two related mouse models, p21^+/Tert^ and p21^+/TertCi^, in which either WT *Tert* or a catalytically inactive *Tert* mutant (TERT^Ci^) is expressed under the control of the *Cdkn1a* (p21) promoter. This strategy was designed to establish a tightly regulated feedback loop, enabling telomerase expression specifically in p21-positive cells. Unexpectedly, we found that both Tert and TertCi suppress p21 expression and attenuate cellular senescence either in the lungs of aged mice (Lipskaia et al, 2024) or in the adipose tissue of young obese mice fed a high-fat diet (Braud et al, 2025). These results suggest that Tert can inhibit senescence through mechanisms independent of its canonical telomere-lengthening activity. Overall, the p21^+/Tert^ and p21^+/TertCi^ models promote Tert expression in pre-senescent cells and facilitate senescence bypass, an early event frequently associated with tumor initiation, particularly in the liver. In this study, we demonstrate that 15% of p21^+/Tert^ and p21^+/TertCi^ mice develop liver tumors by 18 months of age, exhibiting characteristics closely resembling those of human HCC. Through these two mouse models, we identify a telomere-independent role for TERT in liver tumorigenesis and immune modulation. These findings reveal noncanonical Tert functions and highlight molecular and metabolic biomarkers relevant to HCC pathogenesis and immunotherapy response.

## Results

### p21^+/Tert^ and p21^+/TertCi^ mice develop HCCs with features of human HCC

**Figure 1.**
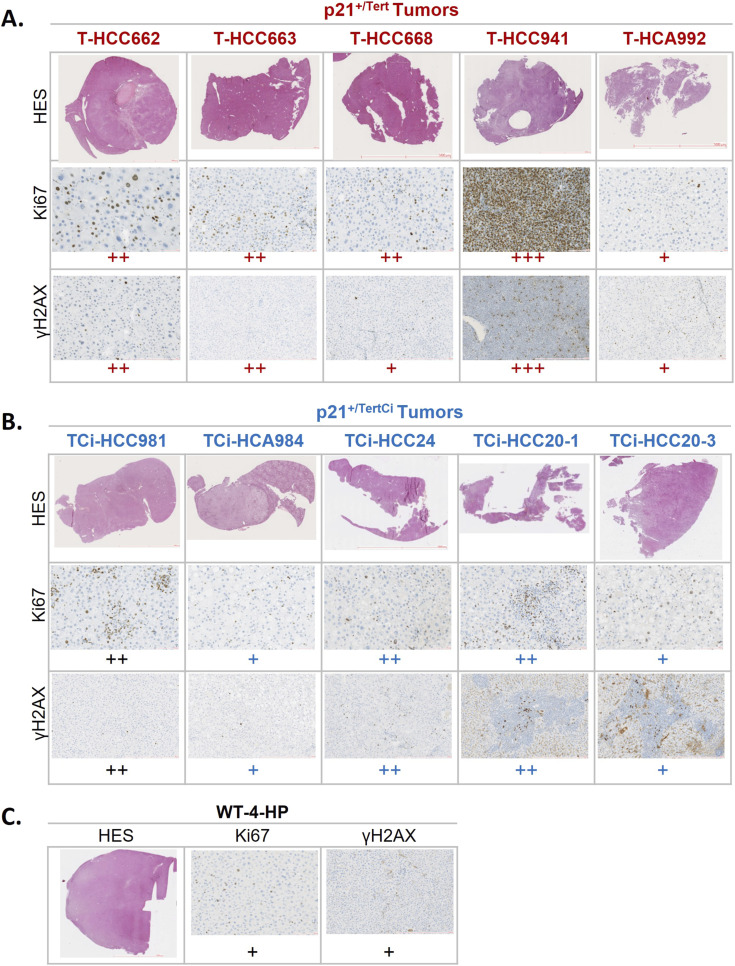
Liver tumors collected from p21^+/Tert^ and p21^+/TertCi^ mice. The histological characteristics of these tumors are summarized in Table 1. **(A, B, C)** Tumor sections were stained with anti-Ki67 and anti-gH2AX antibodies for p21^+/Tert^ tumors (A), p21^+/TertCi^ tumors (B), and WT (C). WT-4-HP represents a case of nodular hepatocellular hyperplasia obtained from an aged WT mouse.

We found that by 18–20 mo of age, both p21^+/Tert^ and p21^+/TertCi^ mice predominantly developed liver tumors. We were surprised to find that mice expressing the catalytically inactive TertCi developed liver tumors with similar frequency to those expressing active telomerase. Most of these tumors were of hepatocellular origin and identified as HCCs or hepatocellular adenomas (HCA). A number of hematological malignancies and liver hemangiosarcomas were also observed (Table 1, Fig 1).

**Table 1.** Histological characterization of tumors.

Genotype	Mouse	Diagnosis	Main histological features
p21+/Tert	662	Liver, hepatocellular carcinoma	Moderately differentiated with solid growth pattern
	663	Liver, hepatocellular adenoma	Moderate cytoplasmic vacuolation
	668	Liver, hepatocellular carcinoma	Well differentiated with trabecular to solid growth pattern
	992	Liver, hepatocellular adenoma	Monomorphic population of neoplastic hepatocytes with numerous small, optically empty vacuoles
	941	Liver, hepatocellular carcinoma	Well differentiated with trabecular to solid growth pattern and multifocal areas of cytoplasmic vacuolation
	995	Liver, hepatocellular carcinoma	Moderately differentiated with solid growth pattern
	985	Liver, hemangiosarcoma	Malignant endothelial tumor with multiple vascular spaces and thrombosis
	861	Liver, hematological malignancy	Diffuse sheets of non-hepatocytic round cells effacing tissue architecture
	975	Liver, hematological malignancy	Diffuse sheets of non-hepatocytic round cells effacing tissue architecture
	944	Liver, hematological malignancy	Diffuse sheets of non-hepatocytic round cells effacing tissue architecture
	861	Colon, diffuse large cell lymphoma	Diffuse sheets of neoplastic lymphoid cells effacing tissue architecture
p21+/TertCi	981	Liver, hepatocellular carcinoma	Well differentiated with trabecular to solid growth pattern
	984	Liver, hepatocellular adenoma	Marked diffuse cytoplasmic vacuolation
	20-1	Liver, hepatocellular carcinoma	Well differentiated with trabecular growth pattern
	20-3	Liver, hepatocellular carcinoma	Well differentiated with trabecular growth pattern
	24	Liver, hepatocellular carcinoma	Well differentiated with trabecular to solid growth pattern
	985	Liver, hemangiosarcoma	Malignant endothelial tumor with multiple vascular spaces and thrombosis
WT	WT4	Liver, non-regenerative nodular hepatocellular hyperplasia	Nodular proliferation of non-neoplastic hepatocytes that retains lobular architecture and without evidence of liver damage
	WT5	Liver, hemangiosarcoma	Malignant endothelial tumor with multiple vascular spaces and thrombosis

We focused our analysis on hepatocellular tumors, given their predominance. HCCs in p21^+/Tert^ and p21^+/TertCi^ mice exhibited histomorphological features reminiscent, at least to some extent, of human HCCs. In general, we noted in most liver tumors, as is the case for human HCC, a nodular to multi-nodular hepatocellular neoplastic lesion with loss of lobular architecture leading to destruction and rarefaction of adjacent parenchyma, although overt tumor invasion was more difficult to identify (Table 1). The murine HCCs were not encapsulated. We found that the growth pattern of HCCs in the transgenic mice was most often “micro-trabecular” (neoplastic hepatocytes forming multiple-cell-thick trabeculae alternating with sinusoidal capillaries), or “solid” (continuous range of neoplastic hepatocytes with very little tumor stroma), with a frequent combination of both patterns within the same tumor (Table 1). These observations are in agreement with the histopathological aspects of human HCC, in which both growth patterns can be found alone or in association. Likewise, the liver tumors from p21^+/Tert^ and p21^+/TertCi^ mice showed multifocal areas of necrosis, hemorrhage, or angiectasia. Of note, other tumor growth patterns described in humans (pseudoglandular, macrotrabecular, scirrhous, etc.) were not observed in the p21^+/Tert^ and p21^+/TertCi^ models. Tumor hepatocytes in both mouse models remained well-differentiated (mimicking mature hepatocytes) with a central round euchromatic nucleus and moderately abundant to markedly abundant granular eosinophilic cytoplasm (Table 1), thus resembling low-grade human HCCs. Accordingly, cellular atypia was mostly mild to moderate with increased nuclear–cytoplasmic ratio, anisokaryosis, and occasional karyomegaly and nuclear pleomorphism. In some tumors, neoplastic hepatocytes showed prominent cytoplasmic vacuolation. Overall, the malignant hepatocellular tumors developed in p21^+/Tert^ and p21^+/TertCi^ mouse models were well-to-moderately differentiated HCCs with trabecular and/or solid growth patterns. Finally, in humans, the adjacent non-tumor liver will be cirrhotic in 80–90% of HCC cases. In our mouse models, hepatic fibrosis of the adjacent parenchyma was minimal to mild, indicating a notable difference from the human disease and its pathogenesis. It is worth noting that hepatocellular hyperplasia was observed in the liver of a WT mouse (designated WT4), and was characterized alongside the liver tumors found in p21^+/Tert^ and p21^+/TertCi^ mice (Fig 1).

To assess tumor cell proliferation, we examined Ki67 expression, a well-established proliferation marker. In human HCC, Ki67 levels are associated with aggressive, highly proliferative tumors (Ramos-Santillan et al, 2024). Consistent with this, all tumors in our study showed markedly increased Ki67 staining compared with normal liver tissue (Fig 1 [Schaeffer et al, 2023]). T-HCC941 exhibited strong Ki67 labeling, reflecting a highly proliferative phenotype, whereas T-HCA992 and TCi-HCA984, both classified as HCA, showed minimal Ki67 labeling (Fig 1). All remaining tumors exhibited high Ki67 levels. We also assessed the levels of gH2AX, a marker of DNA double-strand breaks (DSBs) and genomic instability. Elevated DSBs have been reported in neoplastic lesions of HCC (Xiao et al, 2014). We observed high gH2AX staining in T-HCC662 and T-HCC941, and in both tumors the extent of gH2AX labeling paralleled Ki67 expression (Fig 1). Elevated gH2AX levels were also detected in TCi-HCC20-1 and TCi-HCC20-3, two HCC tumors arising in the same mouse (see below).

Together, our results show that aged p21^+/Tert^ and p21^+/TertCi^ mice (>18 mo) develop well-differentiated liver tumors, predominantly HCC. The ability of both active and inactive Tert, when expressed under the *p21* promoter, to promote tumor formation suggests that Tert contributes to hepatocarcinogenesis also through telomere-independent mechanisms.

**Figure S1.**
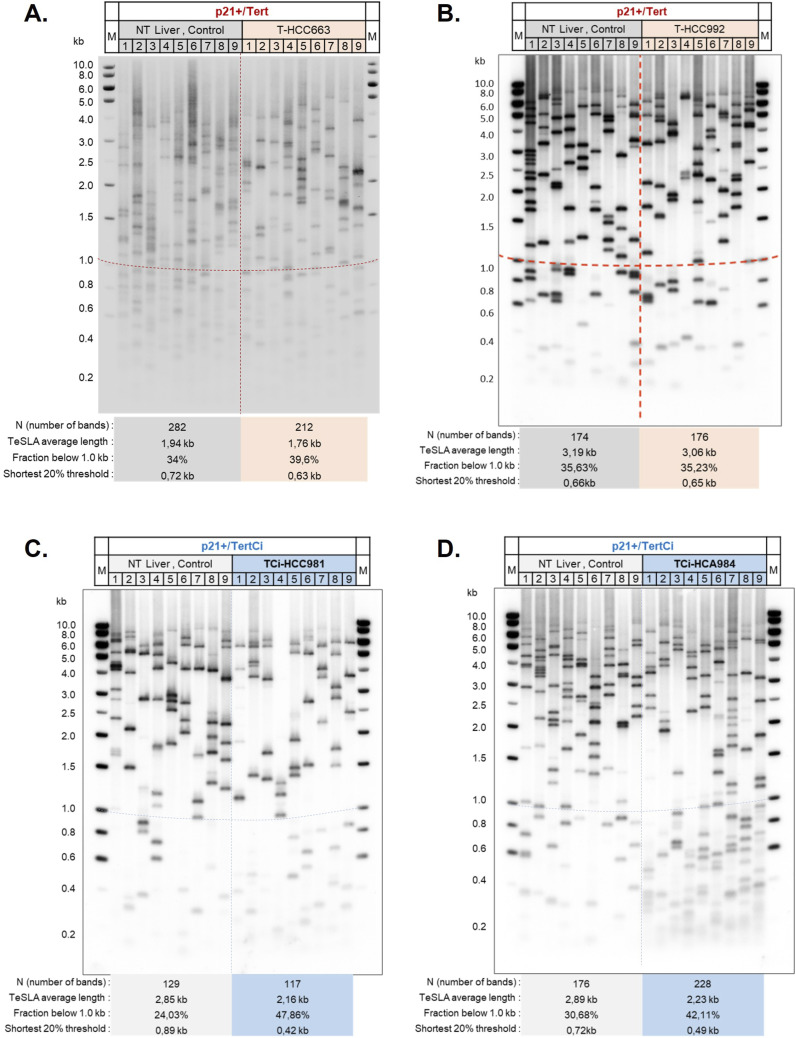
TeSLA p21^+/Tert^ and p21^+/TertCi^ liver tumors. **(A, B)** Quantification of the short telomere fraction in non-tumoral and tumor tissues from T-HCC663 and (B). T-HCA992 using the Telomere Shortest Length Assay (TeSLA). The assay was initiated with 50 ng of genomic DNA. In the final PCR step, 500 pg of ligation product was used per reaction, and nine independent PCR reactions were performed per sample (each lane represents one reaction) to ensure detection of >100 telomeres for accurate quantification. Southern blots were probed for TTAGGG repeats (n = 3 per group). Same analysis as in (A) performed for (C). TCi-HCC981 and (D). TCi-HCA984.

The *TertCi* allele harbors a well-characterized point mutation (D702A) that abolishes telomerase catalytic activity (Choi et al, 2008). To determine whether telomere length differs between p21^+/Tert^ and p21^+/TertCi^ mice, we performed TeSLA (see the Materials and Methods section), which enables the detection of individual critically short telomeres within the bulk telomere population. We analyzed tumors from two p21^+/Tert^ mice (T-HCC663 and T-HCA992; Fig S1A and B) and two p21^+/TertCi^ mice (TCi-HCC981 and TCi-HCA984; Fig S1C and D), alongside their corresponding non-tumoral samples. As shown in Fig S1, the fraction of telomeres shorter than 1 kb was comparable between control and p21^+/Tert^ samples, whereas p21^+/TertCi^ tumors exhibited an increased proportion of telomeres below 1 kb. These results are in agreement with our previous results, indicating that TertCi^i^ lacks catalytic activity (Lipskaia et al, 2024; Braud et al, 2025).

### Distinct somatic mutation profiles in p21^+/Tert^ and p21^+/TertCi^ tumors

**Figure 2.**
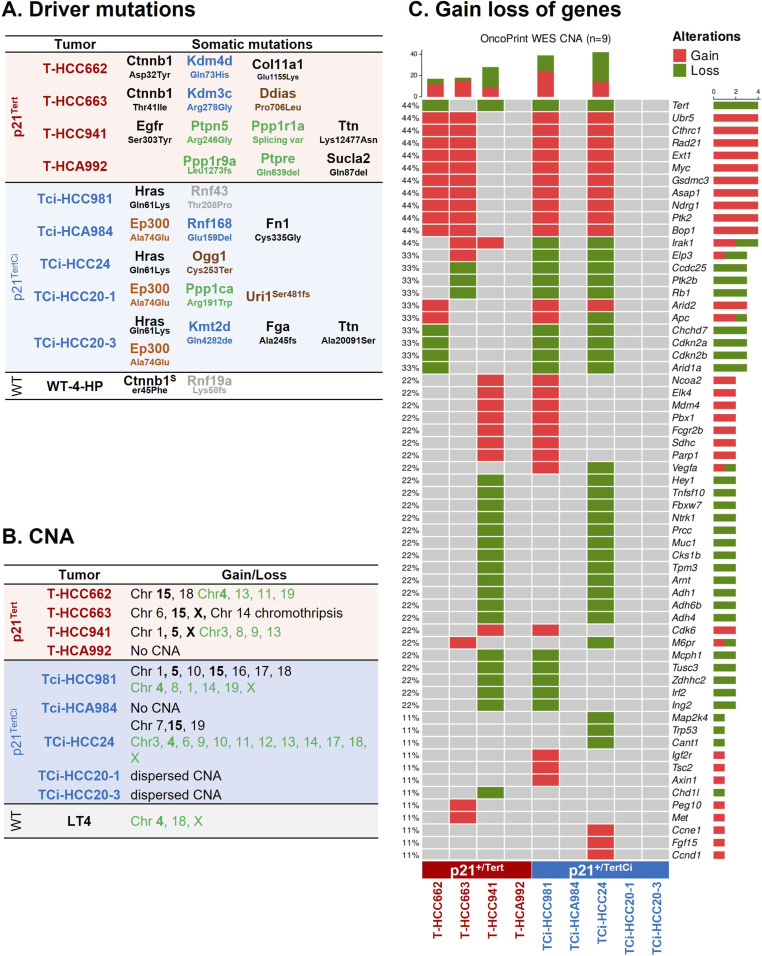
Mutational landscape of liver tumors from aged p21^+/Tert^ and p21^+/TertCi^ mice. **(A)** Somatic mutations in genes found altered in HCC at low or high frequency are indicated. All mutations detected in each tumor are listed in Table S1. **(B)** Copy number alterations in the liver tumors. Chromosomes with gains and loss are indicated in red and green, respectively (see Table S2). **(C)** Genes recurrently amplified or deleted across tumors.

We characterized the mutational landscape of liver tumors from aged p21^+/Tert^ and p21^+/TertCi^ mice using whole-exome sequencing. This analysis identified coding mutations in driver genes and copy number alteration (CNA) (Tables S1 and S2). We examined somatic mutations associated with HCC in both humans and mice (Fig 2A, Table S1, Supplemental Data 1) (Kan et al, 2013; Zhang et al, 2014; Totoki et al, 2014; Letouzé et al, 2017; Connor et al, 2018; L. Chen et al, 2024; J.-S. Lee 2015). In liver tumors from aged p21^+/Tert^ mice, T-HCC662 and T-HCC663 carried activating mutations in *Ctnnb1*, which encodes β-catenin (*Ctnnb1*^*Asp32Tyr*^ and *Ctnnb1*^*Thr41Ile*^, respectively) (Fig 2A). T-HCC662 also harbored mutations in *Col11a1* (*Glu1155Lys*), present in ∼6.8% of human HCCs (Kan et al, 2013), and in the histone demethylase *Kdm4d* (*Gln73His*). T-HCC663 carried mutations in the lysine demethylase *Kdm3c* (*Arg278Gly*) and *Ddias* (*Pro706Leu*), a gene linked to DNA damage-induced apoptosis and elevated in HCC (Im et al, 2023). Tumor T-HCA992 showed few mutations and no CNAs but contained two frameshift mutations: one in *Ppp1r9a* (*Leu1273Thrfs*), a regulatory subunit of protein phosphatase 1 (PP1), and another in *PtprE* (Protein tyrosine phosphatase receptor type E). T-HCA992 also had a truncating mutation in *Sucla2* (*Gln87del*), a TCA cycle enzyme. T-HCC941 harbored a missense mutation in the Furin-like domain of *Egfr* (*Egfr*^*Ser303Tyr*^), a region known to harbor MAPK-activating mutations. Mutations at nearby codon 254 in this domain were frequently found in a carcinogen-induced mouse model of liver cancer (Connor et al, 2018). It also carried a splice-site mutation in *Ppp1r1a* (another PP1 subunit) and a *Ptpn5* (*Arg246Gly*) mutation, encoding PP5 (Protein Phosphatase 5) (Fig 2A).

Table S1. List of mutations identified across the different tumors.

Table S2. Copy number alterations (CNAs) across the different tumors.

Supplemental Data 1. Raw whole-exome sequencing data.

Strikingly, three of five liver tumors from aged p21^+/TertCi^ mice (TCi-HCC981, TCi-HCC20-3, and TCi-HCC24) harbored the same activating *Hras* mutation (*Hras*^*Gln61Lys*^) (Fig 2A), a known hotspot in diethylnitrosamine (DEN)-induced murine liver tumors (Connor et al, 2018). TCi-HCC981 also carried a missense mutation in Rnf43 (*Rnf43*^*Thr208Pro*^), an E3 ubiquitin ligase. TCi-HCC20-3 harbored multiple mutations, including a C-terminal deletion in the histone methyltransferase *Kmt2d* (*Kmt2d*^Gln4282del)^, which mediates H3K4 monomethylation at enhancers (Lee et al, 2013), a frameshift mutation in *Fga* (*Fga*^Ala245fs^), and a missense mutation in Ep300 (*Ep300*^Ala74Glu^). TCi-HCC20-1, which arose in the same mouse as TCi-HCC20-3, harbored the same *Ep300*^Ala74Glu^ mutation but lacked the *Hras*^*Gln61Lys*^ mutation (Fig 2A). TCi-HCC20-1 also carried a mutation in *Ppp1ca* (*Ppp1ca*^*Arg191Trp*^), the catalytic PP1 subunit, as well as a frameshift mutation in *Uri1* (*Uri1*^*Ser481fs*^), a transcriptional repressor implicated in ROS suppression (Xu et al, 2021). TCi-HCC24, which showed extensive genomic rearrangement (Fig 2B), had a truncating mutation in *Ogg1* (*Ogg1*^*Cys253Ter*^), involved in oxidative DNA damage repair (Boiteux & Radicella, 2000). TCi-HCA984, classified as a hepatocellular adenoma, also carried the *Ep300*^*Ala74Glu*^ mutation, marking it as the third tumor with this recurrent mutation. It also harbored a deletion in *Rnf168* (*Rnf168*^*Glu159Del*^), a DSB-repair E3 ligase, and a missense mutation in *Fn1* (*Fn1*^*Cys335Gly*^), which encodes fibronectin (Fig 2A).

Interestingly, mutations in PP1 subunits were identified in multiple tumors, *Ppp1ca* in TCi-HCC20-1, *Ppp1r9a* in T-HCA992, and *Ppp1r1a* in T-HCC941 (Fig 2A, Table S1), highlighting a potential role of the PP1 pathway dysregulation in HCC development (see the Discussion section).

### Shared chromosomal alterations between p21^+/Tert^ and p21^+/TertCi^ tumors mirror human HCC

**Figure S2.**
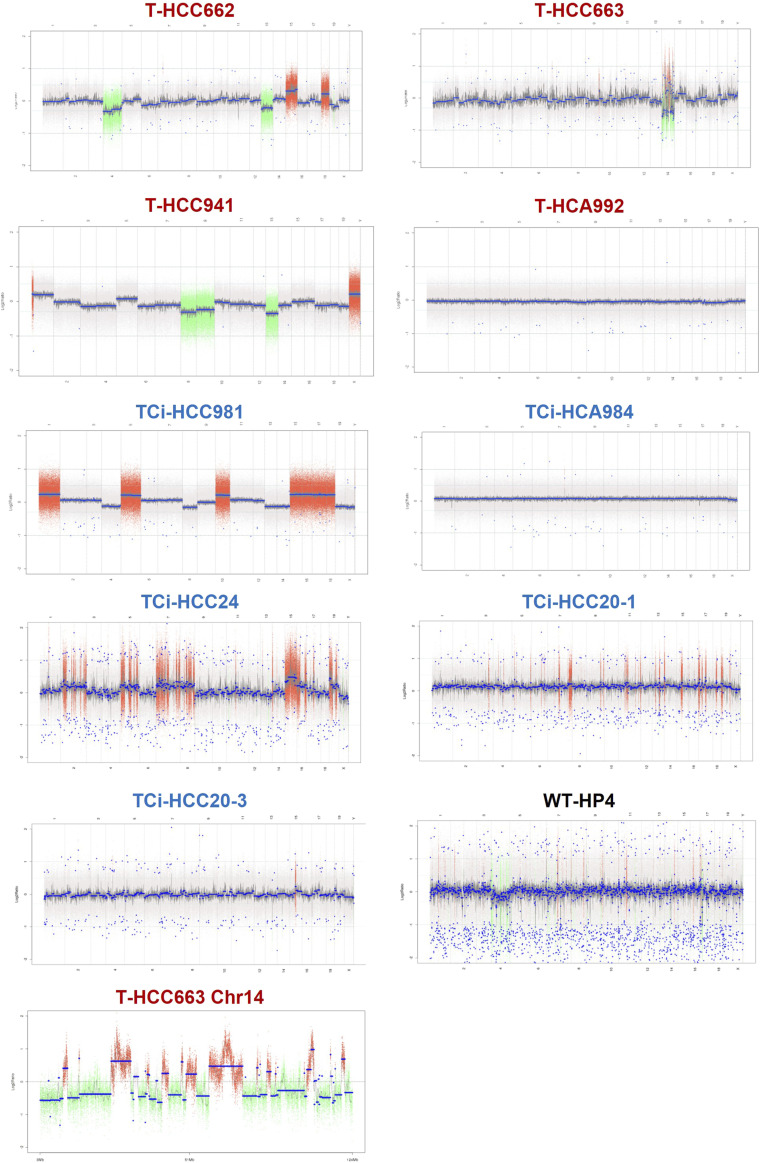
Copy number alteration (CNA) profiles in p21^+/Tert^ and p21^+/TertCi^ liver tumors. Gains and losses are indicated in red and green respectively. Tumors are indicated in the figure and chromosomes are numbered for each tumor.

We analyzed CNAs across the different tumors (Fig 2B, Table S2, Fig S2). No detectable gains or losses were found in T-HCA992 and TCi-HCA984, and minimal alterations in TCi-HCC20-1 and TCi-HCC20-3, in contrast to all other tumors (Fig S2). Recurrent amplifications included a Chr 15 gain in T-HCC662, TCi-HCC981, and TCi-HCC24, a ChrX gain in T-HCC663 and T-HCC941, and a Chr5 gain in T-HCC941 and TCi-HCC981 (Fig 2B). With respect to chromosomal losses, T-HCC662, TCi-HCC981 and TCi-HCC24 showed a loss on Chr 4, often accompanied by a loss on Chr X. A similar pattern of Chr4 and ChrX loss was also detected in the WT liver neoplasm (WT4) (Fig 2B). Interestingly, T-HCC663 showed extensive rearrangement of Chr 14 (Fig S2), indicative of chromothripsis, a catastrophic chromosomal shattering and reassembly event previously reported in ∼5% of human HCC cases (Fernandez-Banet et al, 2014; Chen et al, 2024).

We compiled a curated list of genes recurrently amplified or deleted in human HCC (Zucman-Rossi et al, 2015; Schulze et al, 2016; Niu et al, 2016; Letouzé et al, 2017; Yang et al, 2023; L. Chen et al, 2024) and found strong parallels in the mouse tumors (Fig 2C). In particular, the conserved amplification on Chr 15 includes several key genes (*Myc*, *Cthrc1*, *Rad21*, *Ndrg1*, and *Ptk2*) linked to liver tumorigenesis, tumor growth, metastasis, or immune evasion (Dhanasekaran et al, 2022; Sun et al, 2024; Pang et al, 2024; Tang et al, 2023; Fan et al, 2019). In addition, amplifications include *Met* in T-HCC662 and *Ccnd1* and *Fgf15* (the murine ortholog of human *FGF19*) in TCi-HCC24, frequently amplified in human HCC (Schulze et al, 2015). Chr 5 gains in T-HCC941 and TCi-HCC981 includes *Sdhc* and *Parp1*, genes implicated in HCC progression (Bai et al, 2022; Paturel et al, 2022). Both tumors also showed *Mdm4* amplification, a negative regulator of TP53 found overexpressed in fibrolamellar HCC, particularly in adolescent patients whose tumors often lack TP53 mutations (Karki et al, 2019) (Fig 2C). Among genes affected by copy number loss, only TCi-HCC24 harbors a *Tp53* deletion. *Cdkn2a*, *Cdkn2b*, and *Ard1a* (Chr 4) on their side are deleted in T-HCC662, TCi-HCC981, and TCi-HCC24, whereas T-HCC662, TCi-HCC981, and TCi-HCC24 share a deletion of cell cycle regulators *Cdc25* and *Rb1*. Loss of these genes is linked to senescence bypass and liver tumorigenesis (Zucman-Rossi et al, 2015; Niu et al, 2016; Joseph et al, 2019; Reimann et al, 2024). T-HCC941 and TCi-HCC24 also show deletions in alcohol dehydrogenase genes, including *Adh4*, whose loss correlates with poorer HCC prognosis (Zhang et al, 2023) (Fig 2C). Unlike human HCC, where *TERT* is frequently activated, about half of the tumors exhibited loss of the endogeneous *Tert* locus, likely due to the mouse models used in which *Tert* or *TertCi* are expressed under the control of the *p21* promoter, rendering activation of the native locus dispensable.

In conclusion, despite the limited sample size, our findings show that both *p21*^*+/Tert*^ and *p21*^*+/TertCi*^ mouse models, which mimic telomerase activation in pre-senescent cells, develop HCCs with key molecular and histopathological features of human liver cancer.

### Activating RAS mutations in p21^+/TertCi^ tumors are associated with elevated mutagenesis

**Figure 3.**
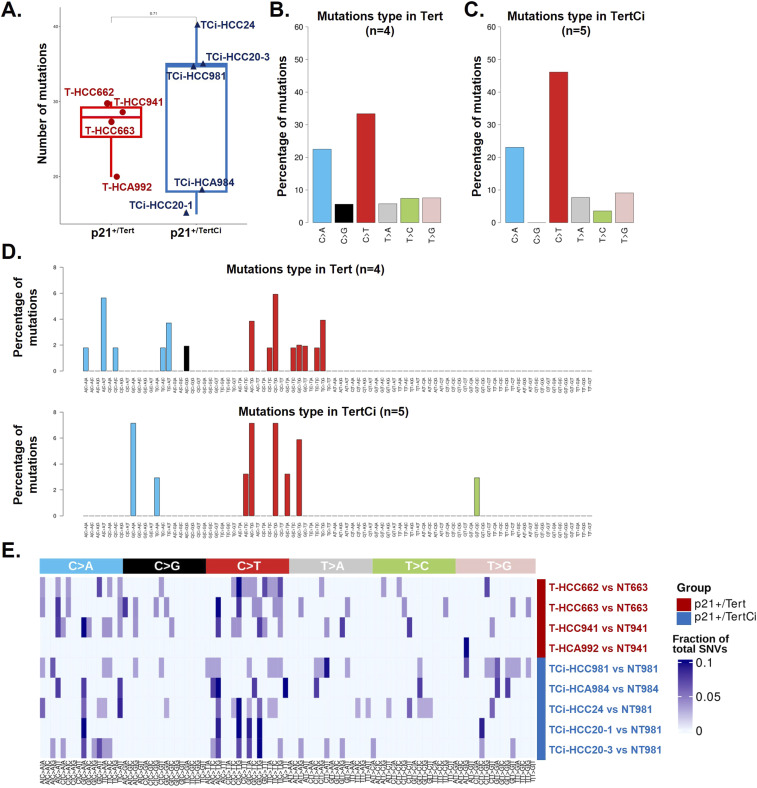
Mutational signature analysis of p21^+/Tert^ and p21^+/TertCi^ tumors. **(A)** Number of somatic mutations per exome in the tumors. **(B, C)** Mutation type in p21^+/Tert^ and p21^+/TertCi^ tumors. **(D)** Occurrence of mutations from p21^+/Tert^ and p21^+/TertCi^ tumor samples classified by substitution type and trinucleotide context. **(E)** Heat map showing mutational profiles of individual mouse tumor samples (rows), categorized by substitution type and trinucleotide context (columns).

We identified recurrent mutations in *Hras*^*Gln61Lys*^ (TCi-HCC981, TCi-HCC24, TCi-HCC20-3) and *Ep300*^*Ala74Glu*^ (TCi-HCA984, TCi-HCC20-1, TCi-HCC20-3) in p21^+/TertCi^ tumors. Notably, in a (DEN)-induced mutagenesis model, half of all liver tumors harbored *Hras* mutations at position Gln61, with *Hras*^*Gln61Arg*^ being the most frequent (Connor et al, 2018). We examined the number and pattern of mutations observed across tumors (Fig 3A). Among p21^+/Tert^ tumors, T-HCC662, T-HCC663, and T-HCC941 exhibited high levels of mutations, whereas T-HCA992 was sparsely mutated (Fig 3A). p21^+/TertCi^ Tumors harboring the *Hras*^*Gln61Lys*^ mutation exhibited a higher number of mutations, whereas the two tumors (TCi-HCA984 and TCi-HCC20-1) with the *Ep300*^*Ala74Glu*^ displayed a lower mutation burden. Interestingly, p21^+/TertCi^ *Hras*^*Gln61Lys*^ tumors display a higher mutation rate than the T-HCC941 *Egfr*^*Ser303Tyr*^ tumor, despite the latter showing markedly elevated Ki67 expression (Fig 1), suggesting that *Hras*^*Gln61Lys*^ may exert a mutator effect.

Mutational signatures analysis in p21^+/Tert^ and p21^+/TertCi^ tumors revealed that C>A transversions (20% of the mutations) occurred at similar overall frequencies in both tumor types, whereas C>T transitions were slightly more frequent (35% and 45% in *p21*^*+/Tert*^ and *p21*^*+/TertCi*^ tumors, respectively) (Fig 3B). C>T transitions occurred preferentially in similar contexts, A[C>T]G and C[C>T]G, in both types of tumors (Fig 3C). Interestingly, in p21^+/Tert^ tumors, C>A transversions predominantly occurred in A[C>A]T and T[C>A]T contexts, whereas in p21^+/TertCi^ tumors, they were mainly enriched in G[C>A]A and T[C>A]T contexts (Fig 3D). The oncogenic mutations *Egfr*^*Ser303Tyr*^ (TCA/TAT) and *Ep300*^*Ala74glu*^ (GCA/GAA) arose in a such context. At the individual tumor level, C>A transversions were enriched in T-HCC941, TCi-HCC981, TCi-HCC24, and TCi-HCC20-3, all harboring activating RAS mutations (Fig 3E). Oddly, T-HCA992 showed no evidence of mutagenesis apart from a few isolated T>G. The few mutations in T-HCA992 are in-frame deletions or frameshift variants.

### Oncogenic signaling pathways in p21^+/Tert^ and p21^+/TertCi^ tumors

**Figure S3.**
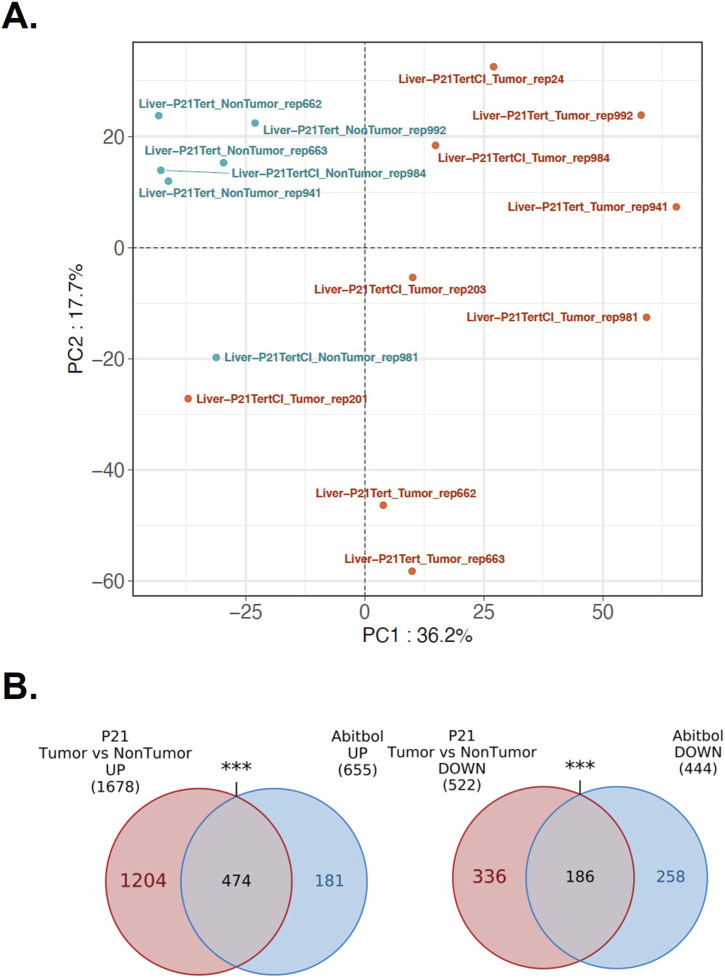
Principal component analysis (PCA) of tumor transcriptomes. **(A)** Principal component analysis (PCA) of tumor transcriptomes. PCA plots showing the clustering of liver tumors based on RNA-seq gene expression profiles. Each point represents an individual tumor sample, highlighting similarities and differences in global transcriptional patterns. **(B)** Venn diagram showing the number and overlap of significantly (*P* < 0.05) up-regulated genes (left panel) or down-regulated genes (right panel) between p21^+/Tert^/p21^+/TertCi^ tumors and HCC carrying an Axin1 deletion (Abitbol et al, 2018).

**Figure 4.**
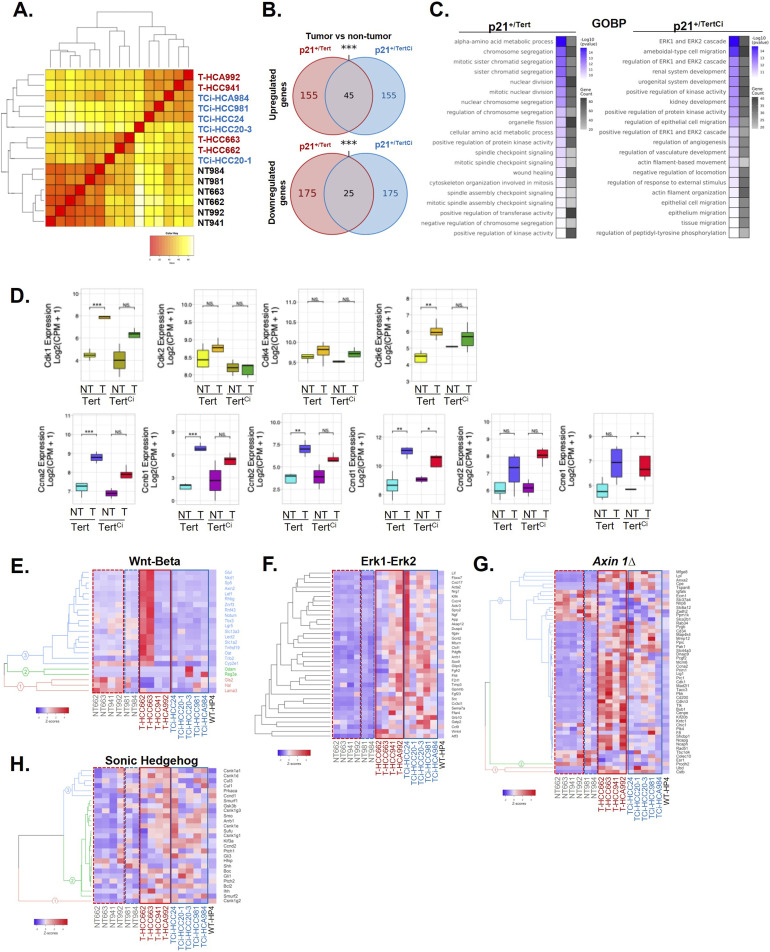
Transcriptomic analysis of p21^+/Tert^ and p21^+/TertCi^ tumors. **(A)** Unsupervised tumor clustering. **(B)** Venn diagram showing the number and overlap of significantly (*P* < 0.05) up-regulated genes (upper panel) or down-regulated (lower panel) between p21^+/Tert^ and p21^+/TertCi^ tumors. **(C)** Gene Ontology Biological Process (GOBP) terms enriched among differentially expressed genes (DEGs) in p21^+/Tert^ (left) and p21^+/TertCi^ (right) tumors. **(D)** Expression levels of cyclin dependent kinases and cyclins in p21^+/Tert^ and p21^+/TertCi^ tumors. Statistics *P < 0.05, **P < 0.01, *P < 0.001 versus NT. **(E, F, G, H)** Heatmaps showing the expression profiles of pathway-specific gene signatures, including activated β-catenin, MAPK–ERK, *axin1Δ*, and activated Sonic Hedgehog pathways, in p21^+/Tert^ and p21^+/TertCi^ tumors. β-catenin and *axin1Δ* signatures were defined in Abitbol et al (2018).

We performed RNA-seq analysis on nine liver tumors, including four from p21^+/Tert^ mice, five from p21^+/TertCi^ mice, one neoplasm from a WT mouse, and six healthy liver samples collected from tumor-bearing mice. Principal component analysis revealed a separation between healthy and tumor samples based on their global gene expression profiles (Fig S3A), highlighting the distinct transcriptional landscape of the tumors. We performed unsupervised tumor clustering (Fig 4A) that shows that T-HCC662, T-HCC663, and TCi-HCC201 segregate into a distinct group. This suggests that TCi-HCC201, harboring the Ep300^Ala74Glu^ mutation, shares transcriptional features with β-catenin–activated tumors. A separate cluster comprises T-HCA92, T-HCC941, TCi-HCA984, TCi-HCC981, TCi-HCC24, and TCi-HCC203. Within this group, T-HCA92 and T-HCC941 align closely with TCi-HCC tumors carrying the hRas^Gln61His^ mutation. Notably, TCi-HCC203, which harbors both *Ep300*^*Ala74Glu*^ and *hRas*^*Gln61His*^, exhibits an intermediate yet distinct transcriptional profile within this cluster.

We identified differentially expressed genes between tumor and healthy liver tissues (Table S3). We found that many of the significantly up- and down-regulated genes were also deregulated in HCCs from the *Axin1Δ* mouse model (Abitbol et al, 2018) (Fig S3B) and in human HCC, underscoring the biological relevance of our models and the presence of conserved HCC markers across species.

Table S3. Transcriptomic (RNA-seq) data from the different tumors.

We next analyzed the 200 most differentially expressed genes in either p21^+/Tert^ or p21^+/TertCi^ tumors compared with non-tumoral liver tissues (Fig 4B). Among these, 45 and 25 genes were commonly up-regulated or down-regulated, respectively, in both tumor types. 155 genes were selectively overexpressed in either p21^+/Tert^ or p21^+/TertCi^ tumors, whereas 175 genes were down-regulated. Gene Ontology analysis of Biological Processes (GOBP) associated with the up-regulated genes revealed distinct patterns between the two models. In p21^+/Tert^ tumors, enriched pathways were primarily related to sister chromatid segregation and mitosis (Fig 4C). In contrast, p21^+/TertCi^ tumors showed a significant up-regulation of genes involved in wound healing, angiogenesis, and the ERK1/ERK2 signaling cascade, aligning with the presence of the *Hras*^*Gln61Lys*^ mutation in three of the five tumors. Consistent with these findings, p21^+/Tert^ tumors overexpress cyclin-dependent kinase 1 (*Cdk1*) and *Cdk6*, along with multiple cyclins (*Ccna2*, *Ccnb1*, *Ccnb2*, *Ccnd1*). p21^+/TertCi^ tumors show a nonsignificant trend toward up-regulation of these genes (Fig 4D).

We next sought to identify transcriptional signatures indicative of signaling pathway activation, specifically Wnt/β-catenin, ERK1/ERK2 (MAPK), Notch, and Sonic Hedgehog (SHH), all of which are frequently implicated in human HCC (Zucman-Rossi et al, 2015). As expected from their mutational profiles (*Ctnnb1*^*Asp32Tyr*^ and *Ctnnb1*^*Thr41Ile*^), T-HCC662 and T-HCC663 displayed strong activation of the Wnt/β-catenin pathway (Fig 4E). These two tumors showed high expression of a ten-gene mutated β-catenin gene signature comprising AXIN2, GLUL, LGR5, NKD1, NOTUM, RHBG, SLC13A3, SP5, TCF7, and TNFRSF19, which has been reported to identify CTNNB1-mutated human HCC (Lehrich et al, 2024). In addition, T-HCC662 and T-HCC663 showed marked overexpression of the Na^+^-dependent amino acid transporters *Slc1a5*, which mediates glutamine uptake and has recently been recognized as a novel hallmark of HCC (Tambay et al, 2024), and *Slc1a2*, which transports glutamate.

Tumors harboring the *Hras*^*Gln61Lys*^ mutation, TCi-HCC24, TCi-HCC20-3, and TCi-HCC981 (all from p21^+/TertCi^ mice), along with T-HCC941 (p21^+/Tert^, carrying *Egfr*^*Ser306Tyr*^), exhibited a shared transcriptional signature consistent with ERK1/ERK2 (MAPK) pathway activation, in alignment with their mutational backgrounds (Fig 4F). Further, T-HCC662, T-HCC663, and T-HCA992 exhibited transcriptional features of Notch pathway activation, similar to those observed in *AxinΔ* mouse models (Villanueva et al, 2012; Abitbol et al, 2018) (Fig 4G). A distinct (SHH) signature was found in TCi-HCC981, TCi-HCA984, T-HCA992, and TCi-HCC24 (Fig 4H), indicating additional heterogeneity in pathway activation across tumors. Overall, the transcriptomic profiles of the tumors are largely consistent with their underlying mutational landscapes. Interestingly, T-HCA992 showed a MAPK activation signature, despite lacking canonical *RAS* pathway mutations. We hypothesize that this activation could result from mutations in phosphatase genes (*PtprE* and *Ppp1r9a*), which may lead to increased substrate phosphorylation and thereby activate MAPK signaling (Chen et al, 2024).

**Figure S4.**
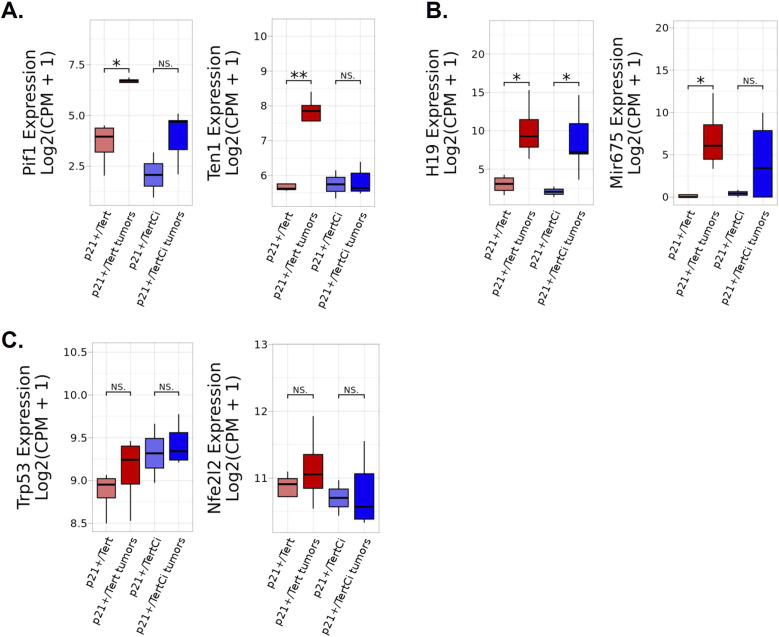
Expression levels in p21^+/Tert^ and p21^+/TertCi^ tumors. **(A)** Pif1 and Ten1. **(B)** H19 and Mir675. **(C)** Nrf2 (Nfe2l2) and Tpr53 Statistics *P < 0.05, **P < 0.01 versus non-tumoral p21^+/Tert^ or p21^+/TertCi^.

Among model-specific transcriptional signatures, only p21^+/Tert^ tumors exhibited overexpression of the helicase Pif1 and Ten1, a component of the CST complex (Ctc1/Stn1/Ten1) involved in telomere replication and stability (F. Wang et al, 2012) (Fig S4A). In addition, nearly all tumors, except TCi-HCA984 and TCi-HCC20-1, showed strong overexpression of the maternally imprinted long noncoding RNA *H19* and its associated microRNA, miR-675, both of which have been implicated in liver cancer pathogenesis (Wang et al, 2023) (Fig S4B).

### Tumors exhibit suppressed gluconeogenesis but up-regulate distinct Nfrf2 target genes

**Figure 5.**
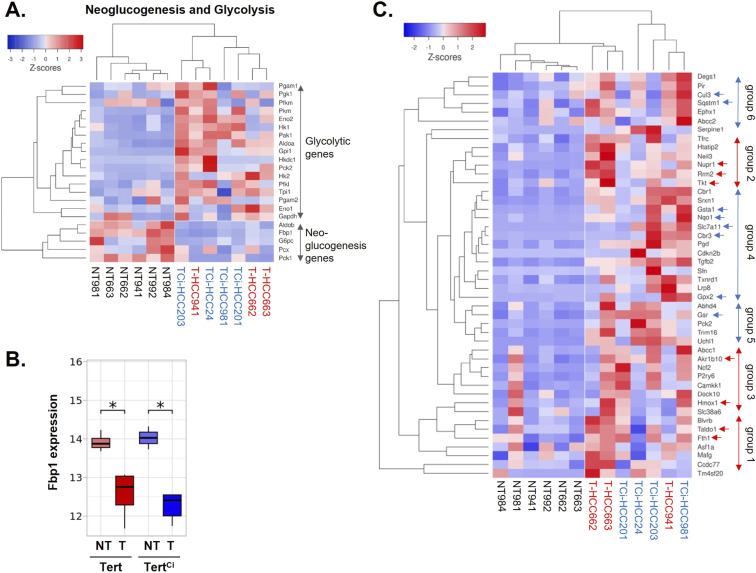
Down-regulation of gluconeogenesis and activation of Nrf2 targets in tumors. **(A)** Heatmap illustrating the expression patterns of genes associated with gluconeogenesis and glycolysis, based exclusively on HCC samples. **(B)** Expression levels of FBP1 in p21^+/Tert^ and p21^+/TertCi^ tumors, Statistics *P < 0.05 versus non-tumoral p21^+/Tert^ or p21^+/TertCi^. **(C)** Heatmap depicting the expression of Nrf2 target genes across HCC samples. Groups 1–3 highlight genes predominantly up-regulated in T-HCC662, T-HCC663, and TCi-HCC201. Groups 4–6 represent genes mainly up-regulated in MAPK-activated tumors. Red and blue arrows indicate genes associated with resistance to ferroptosis (see text).

A recent study (Gu et al, 2025) showed that during the progression from metabolic dysfunction–associated steatohepatitis (MASH) to HCC, FBP1 expression declines in premalignant hepatocytes. To explore metabolic alterations in the tumor samples, we analyze the expression of genes involved in glycolysis and gluconeogenesis. To improve interpretability, we restricted our analysis to HCC tumors (Fig 5A). Across all samples, we observed consistent down-regulation of the gluconeogenic genes *AldoB* and *Fbp1* (Fig 5A and B). Notably, Fbp1 has been shown to form a stable complex with AldoB, Pp2A, and Akt, leading to Akt inactivation (Gu et al, 2023). Regarding glycolytic genes, *hRas*^*Gln61His*^ TCi-HCC203, T-HCC941, and TCi-HCC24 exhibit broad up-regulation of a large set of glycolytic genes (*Pgam1*, *Pgk1*, *Pfkm*, *Pkm*, *Eno2*, *Hk1*, *Pak1*, *AldoA*, *Gpi1*, *Hkdc1*, *Pck2*, *Pfl*, and *Tpi1*), whereas the remaining tumors display increased expression of a more limited subset, including *Hk2*, *Tpi1*, *Eno1*, and *Gapdh*, which are consistently overexpressed across T-HCC662, T-HCC663, and TCi-HCC201 (Fig 5A).

Because *Fbp1* down-regulation has been linked to NRF2 activation (Gu et al, 2025), we examined the expression of Nrf2 (Nfe2l2) target genes (Malhotra et al, 2010; Mitsuishi et al, 2012b) in HCC. We found that most Nrf2 targets involved in redox and energy metabolism regulation (Luchkova et al, 2024) were differentially regulated in tumor samples (Fig 5C). Clustering analysis did not reveal a tumor model–specific signature. Instead, it identified groups of genes commonly expressed across subsets of tumors that distinguish β-catenin–activated tumors from hRAS-activated tumors. Specifically, groups 1 and 2 are predominantly expressed in T-HCC662 and T-HCC663, whereas Group 3 genes are mainly observed in T-HCC663 and TCi-HCC201, somewhat consistent with tumor clustering shown in Fig 4A. Group 4 genes are primarily overexpressed in TCi-HCC203, T-HCC941, and TCi-HCC981, as well as in T-HCC663, TCi-HCC24, and TCi-HCC203.

The expression profiles point to a coordinated ferroptosis-protective program driven by distinct sets of genes. In Fig 5C, genes associated with increased resistance to ferroptotic cell death are highlighted: those mainly overexpressed in groups 1, 2, and 3 are shown in red (*HmoX1*, *Fth1*, *Taldo1*, *Tm4sf20*, *Akr1b10*, *Tkt*, *Rrm2*, *Nupr1*). These genes include *Tkt* (transketolase) and *Taldo1* (transaldolase 1) involved in the pentose phosphatase pathway as well as *Nupr1*, a stress responsive gene induced under oxidative and metabolic stress and known to be expressed in human HCC (Huang et al, 2021; Cai et al, 2025) (Fig 5C). Genes preferentially overexpressed in groups 4, 5, and 6 are shown in blue (*Slc7a11*, *Gpx2*, *Gsr*, *Nqo1*, *Cbr3*, *Gsta1*, *Cul3*, *Sqtsm1*, and *Gpx2*). Many of these genes are regulators of ferroptosis resistance, a cell death pathway driven by lipid peroxidation (Koppula et al, 2021; Su et al, 2025) (Fig 5C). Although there is some redundancy between the tumors, these results suggest that β-catenin tumors and TCi-HCC201 resist ferroptosis primarily by limiting free iron and boosting NADPH production, whereas MAPK-activated tumors rely on a canonical NRF2-SLC7A11-glutathione axis to detoxify lipid peroxides through enhanced antioxidant enzyme activity.

When considering all tumors, neither Nrf2 nor Tp53 is significantly overexpressed in tumors (Fig S4C). Notably, although Nrf2 mRNA levels were higher in T-HCA992 and TCi-HCC24 compared with TCi-HCC20-3 and TCi-HCC981, the latter two showed the strongest up-regulation of Nrf2 target genes. This suggests that Nrf2 protein stabilization may drive its activity in the tumors rather than its transcriptional regulation.

### Spatial analysis reveals altered HNF4α protein levels and liver tissue architecture

**Figure S5.**
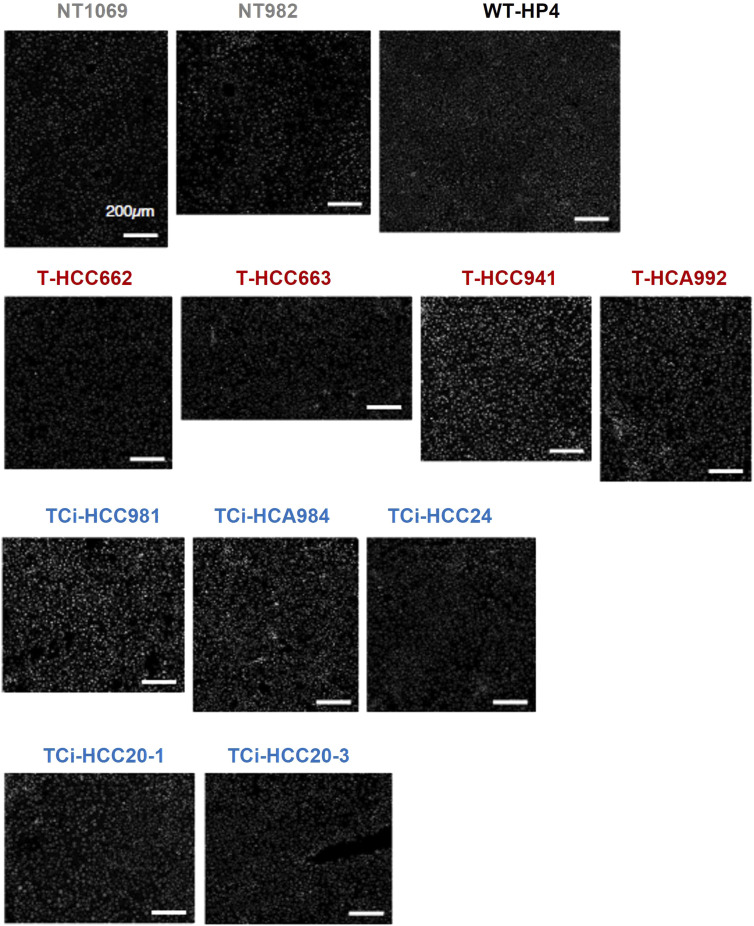
Scale bars for the uncropped images of p21^+/Tert^ and p21^+/TertCi^ liver tissue and corresponding tumors.

**Figure 6.**
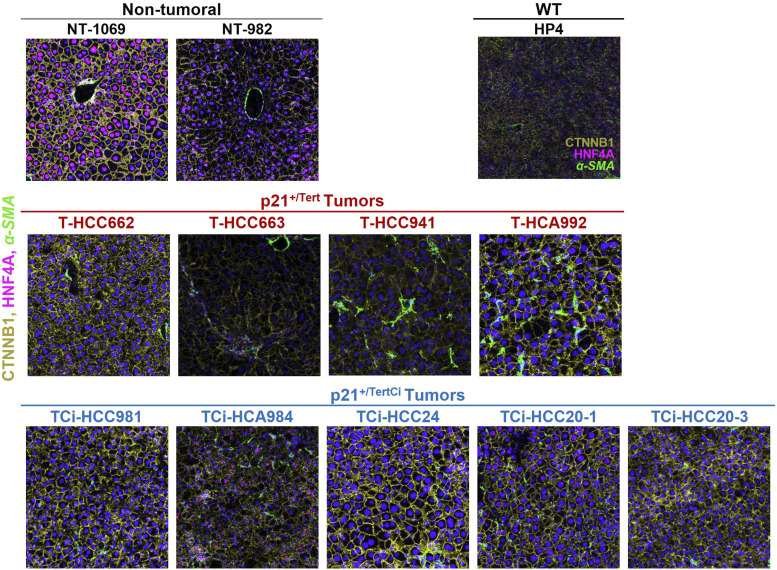
Spatial architecture of liver tumors. Formalin-fixed, paraffin-embedded sections of non-tumoral and tumor tissues were analyzed using the Hyperion Imaging System with antibodies against Ctnnb1 (yellow), Hnf4α (magenta), and α-Sma (green). NT1069 and NT982 denote non-tumoral liver tissues from p21^+^/Tert and p21^+^/^−^ mice, respectively. Tumors are labeled as indicated in the figure and described in Table 1, with the number after each tumor name corresponding to the region of interest. An iridium intercalator was used for nuclear staining. Images are included in the supplemental files. Source data are available for this figure.

We used the Hyperion Imaging System, which enables high-dimensional, subcellular-resolution analysis of multiple cellular targets simultaneously in formalin-fixed paraffin-embedded (FFPE) tissues (Elaldi et al, 2021). Using a validated panel of 10 metal-conjugated antibodies (Table S4), we analyzed the architecture of the tissues. Scale bars for the uncropped images of p21^+/Tert^ and p21^+/TertCi^ liver, as well as the tumors, are provided in Fig S5. To characterize the liver parenchyma, we first stained healthy livers and the tumors from p21^+/Tert^ and p21^+/TertCi^ mice using antibodies directed against β-catenin (Ctnnb1), Hepatocyte nuclear factor 4α (Hnf4α), and alpha Smooth Muscle Actin (α-Sma) (Fig 6). In healthy p21^+/Tert^ (NT1069) and p21^+/TertCi^ (NT982) livers (Fig 6), a majority of cells were stained by HNF4α antibodies likely, marking non-transformed hepatocytes. β-catenin displayed a typical WT distribution with predominant localization at hepatocyte plasma membrane, consistent with its role in cell–cell adhesion and α-SMA was primarily restricted to the perivascular regions where smooth muscle cells reside (Fig 6). It was challenging to distinguish whether 4T1 or T-HCC662 and T-HCC663 tumors, which share an activating mutation in Ctnnb1, exhibited higher levels of cytoplasmic or nuclear β-catenin indicative of β-catenin activation (Fig 6). Notably, all tumors showed a reduced number of Hnf4α-positive cells as well as a decrease in staining intensity (see the following paragraph for HNF4α quantification across tumors). Moreover, unlike the perivascular localization observed in the healthy livers, we observed dispersed α-Sma–positive cells throughout the tumor, likely reflecting the presence of cancer-associated fibroblasts (Sun et al, 2023; Xu et al, 2022). In tumors T-HCC941, T-HCA992, and TCi-HCC20-1, α-Sma–positive areas accounted for more than 20% of the area occupied by DNA, indicating an important stromal activation.

Table S4. Validated markers used for imaging mass cytometry.

**Figure 7.**
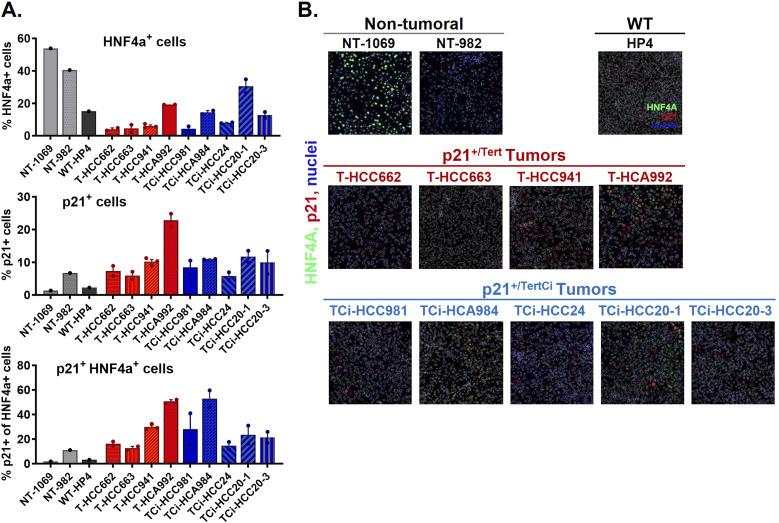
Quantification of HNF4α and p21 levels across tumor samples. **(A)** ROIs from liver tumor sections were imaged using the Hyperion with antibodies against HNF4α and p21. Signal quantification was performed as described in the Materials and Methods. The percentage of HNF4αA, p21, and double-positive cells are shown. The two dots for each tumor represent two different ROI. **(B)** Representative images of non-tumoral and tumoral sections. HNF4α (Green), p21 (Red), and nuclei (Blue). Some samples were used for representative images shown in Figs 7 and 8.

We next quantified Hnf4α and also p21-positive cells across all the samples (Fig 7A and B). In healthy p21^+/Tert^ and p21^+/TertCi^ livers, 40–50% of liver cells expressed Hnf4α. This proportion sharply dropped to 10% or less in the tumors harboring the Ctnnb1^Asp32Tyr^, Ctnnb1^Thr41Ile^, Egfr^Ser303Tyr^, and Hras^Gln61Lys^ mutations (Fig 7A and B). In contrast, T-HCA992, TCi-HCA984, and TCi-HCC201, lacking activation of the Wnt/β-catenin or hRas signaling pathways retained ∼20% of Hnf4α-positive cells (Fig 7A and B). These findings prompted us to examine the transcript levels of HNF4α. RNA-seq analysis did not reveal significant down-regulation of Hnf4α mRNA levels across tumor samples (Table S3), suggesting that HNF4α may be subject to post-transcriptional regulation in tumors with activated Wnt/β-catenin or hRAS signaling.

On the same FFPE sections, we quantified the proportion of p21-positive cells. In NT1069 (p21^+/Tert^) and 4T1 (p21^+/+^) livers, fewer than 2% of cells were p21 positive compared with ∼5% in NT982 (p21^+/TertCi^) healthy liver (Fig 7A and B). This proportion increased to above 10% in T-HCC941, T-HCA992, TCi-HCA984, and TCi-HCC20-1 (Fig 7A and B) and was about 5% for the other tumors, consistent with the transcriptional overexpression of p21 in all tumors (Table S3). One tumor (HCA), T-HCA992, exhibited a markedly higher proportion of p21-positive cells (>20%) (Table S3). Interestingly, in T-HCA992 and TCi-HCA984 both classified as hepatocellular adenoma (Fig 1, Table 1), ∼50% of Hnf4α-positive cells were also positive for p21. Notably, both tumors display high levels of Tert mRNA, suggesting that Tert may drive hepatocyte proliferation in these two tumors, which lack mutations in the Wnt/β-catenin and hRAS pathways.

### Tumor infiltration by immune cells is altered in β-catenin–activated HCC

**Figure 8.**
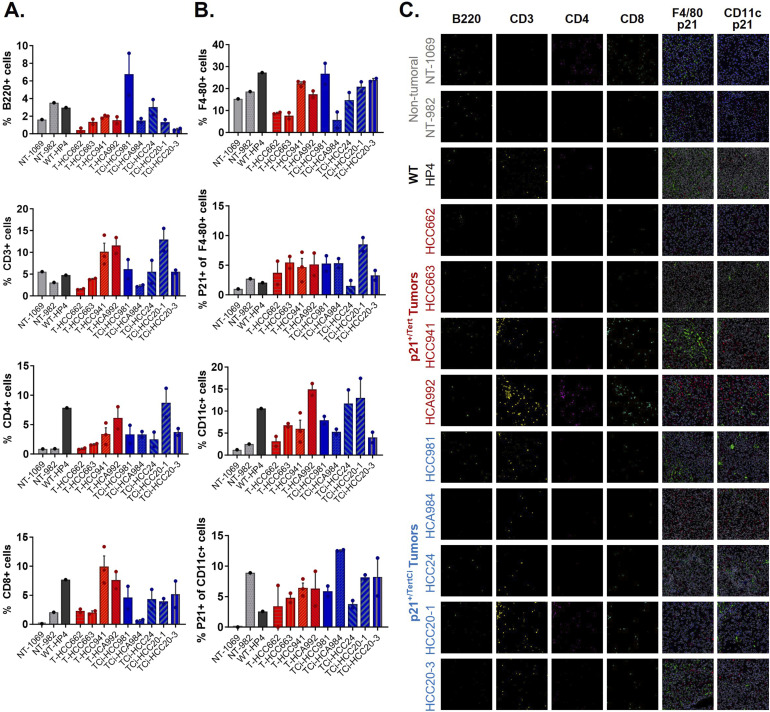
Tumor infiltration by lymphocyte subsets, macrophages, and dendritic cells. **(A)** ROIs were imaged using antibodies against B220, CD3, CD4, CD8 marking B-lymphocytes, T-lymphocytes, auxiliary (helper) T cells, and cytotoxic T cells. **(B)** ROIs were imaged using antibodies against F4/80 (macrophages), CD11c (dentritic cells), and p21 to label macrophages, dendritic cells, and p21.r. Signal quantification was performed as described in the Materials and Methods section. Immune cell infiltration is shown. The percentage of positive cells for the indicated markers is shown for each tumor. **(C)** Representative images of tumor sections: B220 (Green), CD3 (Yellow), CD4 (Magenta), CD8 (Cyan), and nuclei (Blue). F4/80 is shown in green, CD11c in magenta, and nuclei in blue. Mosaic uncropped images are included in the supplemental files. Some samples were used for representative images shown in Figs 7 and 8.

We further assessed immune cell infiltration within the tumors. To characterize the infiltration of lymphocyte classes, we used metal-conjugated antibodies against Cd3 (T lymphocytes), Cd4 (auxiliary T lymphocytes), Cd8 (cytotoxic T lymphocytes), and B220 (B lymphocytes). We found that the two β-catenin–activated tumors (T-HCC662 and T-HCC663) were poorly infiltrated by T-lymphocytes (2–4% of Cd3-positive cells) with 1–2% of auxiliary and cytotoxic T lymphocytes (Fig 8A and C). These results are consistent with previous studies showing that activation of the Wnt/β-catenin pathway in HCC is associated with poor T-lymphocyte infiltration, facilitating immune evasion and resistance to anti-PD1 therapy (Dantzer et al, 2024). In contrast, T-HCA992, TCi-HCC20-1, and T-HCC941 tumors showed a higher level of T lymphocyte infiltration (>10%) (Fig 8A and C). Whereas TCi-HCC20-1 was characterized by a higher proportion of auxiliary (CD4^+^) T cells (8–10%) compared with cytotoxic (CD8^+^) T cells (<4%), the highly aggressive T-HCC941 (Egfr^Ser303Tyr^) displayed a predominance of cytotoxic T cells (∼10%) over auxiliary T cells (Fig 8A and C). TCi-HCC981, TCi-HCC20-3, and TCi-HCC24 tumors, all carrying the Hras^Gln161Lys^ mutation, displayed comparable intermediate levels of lymphocyte infiltration (∼5%). In summary, the two β-catenin–activated tumors exhibited the lowest levels of T-cell infiltration, whereas the Egfr-activated tumor, along with tumors T-HCA992 and TCi-HCC20-1 (which both harbor mutations in subunits of PPP1 exhibited the highest degree of T lymphocyte infiltration [∼5%]). Regarding B lymphocyte infiltration, tumors exhibited very low levels of infiltrating B lymphocytes (1–3%) (Fig 8A and C). Only TCi-HCC981 exhibited a proportion of infiltrating B lymphocytes exceeding 5%.

We further quantified the proportion of macrophages (F4/80) and dendritic cells (Cd11c) in the liver samples (Fig 8B and C). In healthy liver samples (NT1069 and NT982), F4/80-positive cells accounted for ∼15% of total liver cells, likely consistent with their identification as Kupffer cells (Fig 8B and C). This proportion increases to about 25% in the 4T1 neoplastic tissue carrying the *Ctnnb1*^*Se45Phe*^ but not the activated β-catenin signature. The percentage of macrophages in tumors ranged from 5% in minimally infiltrated tumors (T-HCC662 *Ctnnb1*^*Asp32Tyr*^, T-HCC663 *Ctnnb1*^*Thr41Ile*^, TCi-HCA984 *Ep300*^*Ala74Glu*^) to 20–25% in highly infiltrated tumors (T-HCC941 *Egfr*^*Ser303Tyr*^, TCi-HCC981 *Hras*^Gln61Lys^, TCi-HCC20-3 *Hras*^Gln61Lys^), suggesting that the degree of macrophage infiltration is associated with the oncogenic driver and reflects heterogeneity within the tumor microenvironment (TME). Among these macrophages, in both healthy and tumor tissue of aged p21^+/Tert^ and p21^+/TertCi^ mice, only 2–5% were positive for p21, indicating that the vast majority of macrophages do not express p21 (Fig 8B and C). Dendritic cells (Cd11c) infiltration was low in healthy livers (1–2%) and in T-HCC662 and T-HCC663 tumors, which show generally weak immune cell infiltration, as well as in TCi-HCA984 (HCA) (Fig 8B and C). T-HCA992, TCi-HCC24, and TCi-HCC20-1 showed higher levels of dendritic cell infiltration with marked punctuate staining (Fig 8B and C). Of note, dendritic cells exhibited a higher proportion of p21-positive cells than macrophages (Fig 8B and C). Interestingly, tumors such as T-HCC941 and TCi-HCC20-3 tumors, being markedly infiltrated by macrophages (>20%), exhibited low levels of dendritic cells (3–7%), suggesting competitive dynamics between macrophages and dendritic cells in the TME.

## Discussion

The p21^+/Tert^ and p21^+/TertCi^ mouse models develop hepatocellular liver tumors, including HCC and HCA. In our murine models, HCCs resemble human HCCs in many histomorphological aspects, including trabecular and solid growth patterns, multifocal necrosis, hemorrhage, and well-to-moderately differentiated tumor cells with mild to moderate atypia. These features support the translational relevance of these models for studying human HCC.

The finding that conditional expression of TertCi promotes liver tumorigenesis in aged mice raises questions about the mechanism through which TertCi drives tumor development. Because models p21^+/Tert^ and p21^+/TertCi^ retain an intact endogenous *Tert* locus, the possibility of endogenous *Tert* reactivation was considered. However, no mutations were found in the *Tert* promoter region, and CNA analysis suggests a loss rather than amplification of the *Tert* locus (Fig 2C). In addition, our TeSLA experiments suggest that the telomeres in p21^+/TertCi^ tumors are not maintained via ALT. We rather favor the possibility that TERTCi modulates signaling pathways that promotes liver tumor development. This analysis is complex, as most tumors are also associated with oncogenic mutations. It is also possible that a mitochondrial form of TertCi (and Tert) contributes to tumor development by safeguarding mitochondrial DNA and function (Judith et al, 2009; Saretzki 2014) in tumors that are exposed to high oxidative stress. This hypothesis is consistent with the observed overexpression of Src in all tumors, as Src kinase activity has been shown to promote the nuclear export of TERT (Haendeler et al, 2003).

We observed that p21^+/Tert^ tumors were associated with distinct oncogenic mutations. Specifically, the *Ctnnb1*^*Asp32Tyr*^ and *Ctnnb1*^*Thr41Ile*^ variants are both well-characterized exon 3 gain-of-function mutations known to activate Wnt/β-catenin signaling (Rebouissou et al, 2016; Dantzer et al, 2024). Consistent with this, T-HCC662 and T-HCC663 displayed a strong transcriptional signature of β-catenin activation (Fig 4D). In addition to β-catenin mutations, T-HCC662 and T-HCC663 carried mutations in *Kdm4d* (*Jmjd2d*) and *Kdm3c*, respectively, two distinct JmjC H3K9 demethylases. This raises the intriguing question of whether the *Kdm4d*^*Gln73His*^ mutation observed in T-HCC662 might impact β-catenin–driven transcription in this context. Notably, mutations *Kdm4d* and *Kdm3c* were exclusive to T-HCC662 and T-HCC663, suggesting potential roles in Wnt pathway modulation. Surprisingly, T-HCA992 exhibited no genomic rearrangements but did harbor two frameshift mutations in the phosphatases *Ppp1r9a* and *PtprE*. Similar mutations were observed in multiple tumors in this study (Fig 2A, Table S1). Of note, recent work has reported mutations in UTR regions of PP1 subunits in human HCC (L. Chen et al, 2024). PP1 has been shown in human cells to bind AXIN and oppose its phosphorylation (Luo et al, 2007). We observed suppressed Wnt signaling in T-HCA992 (Fig 4D), consistent with the expected effect of PP1 inactivation, which likely increases AXIN1 phosphorylation, enhances GSK3-mediated β-catenin degradation, and thereby dampens Wnt pathway activity (Luo et al, 2007).

T-HCC941 also harbored a splice-site variant in another PP1 regulatory subunit, *Ppp1r1a*, along with a mutation in *Ptpn5* (Arg246Gly), which encodes protein phosphatase 5 (PP5). PP5 inhibition has been shown to suppress HCC growth by activating AMPK signaling (Y.-L. Chen et al, 2017). Interestingly, T-HCC941 also exhibited the AXIN1-associated transcriptional signature, further reinforcing the connection between phosphatase subunit mutations and AXIN1-related pathway dysregulation. These findings underscore the significant potential role of phosphatases in liver cancer development.

Surprisingly, three of five tumors from aged p21^+/TertCi^ mice (TCi-HCC981, TCi-HCC20-3, and TCi-HCC24) shared the activating *Hras*^Gln61Lys^ mutation. Gln61 is a mutation hotspot (*Gln61Lys*, *Gln61Arg*, and *Gln61Leu*) found at high frequency in DEN-induced liver tumors, but absent in spontaneous cases (Connor et al, 2018). TCi-HCC981, TCi-HCC20-3, and TCi-HCC24 displayed the highest level of mutagenesis. The finding that tumors harboring activating RAS mutations exhibit increased mutagenesis was notably evident in the comparison between TCi-HCC20-1 and TCi-HCC20-3, which originate from the same liver and are genetically similar except for the presence of *Hras*^*Gln61Lys*^ in TCi-HCC20-3. Interestingly, T-HCC941 (*Egfr*^*Ser303Tyr*^), despite being the most highly proliferative tumor, exhibited a lower overall mutational burden compared with *Hras*^*Gln61Lys-*^mutated tumors (Fig 3A), raising the possibility of a mutator phenotype associated with mutant hRas. Of note, the Hras^Gln61Lys^ (CAA/AAA) arose from a C>A transversion. In the DEN-induced mouse model, T>A and T>C predominate, reflecting the mutagenic properties of DEN (Connor et al, 2018). In contrast, tumors from the p21^+/Tert^ and p21^+/TertCi^ mice show a higher frequency of C>A and C>T mutations. The predominance of C>T transitions in ACG and CCG contexts (Fig 3E) suggests that the enrichment of C>T mutations in both tumor types may reflect proliferation-associated cytosine deamination likely at CpG dinucleotides (Tate et al, 2019; Alexandrov et al, 2020). On their side, C>A transversions are often linked to oxidative DNA damage. Notably, these transversions occurred predominantly in distinct trinucleotide contexts (ACT and GCA) in p21^+/Tert^ and p21^+/TertCi^ mice, respectively, both contexts being consistent with oxidative stress, but the difference may be linked to the associated oncogenic mutation, reflecting a mechanistic difference in tumor initiation and evolution in these models. Interestingly, TCi-HCC24, which exhibits the highest mutational burden, carries a truncating Ogg1^Cys253Ter^ mutation.

The results raise the question of whether Tert*Ci* expression contributes to the emergence of the Hras^*Gln61Lys*^ (CAA/AAA) mutation through a direct or indirect specific mutagenic effect, or whether it instead reflects enhanced cell survival and proliferation. In our previous work, we found that Tert, but not TertCi, reduces oxidative damage (Lipskaia et al, 2024; Braud et al, 2025). The high mutation burden in *Hras*-driven tumors might be a consequence of the oncogene’s own mutagenic activity. Similarly, TCi-HCA984, TCi-HCC20-1, and TCi-HCC20-3 all harbor the same *Ep300* mutation (Ala74Glu; GCA→GAA), which also arises from a C>A transversion. *EP300* mutations are present in only 2% of HCC cases in the TCGA Pan-Cancer Atlas cohort (n = 366, whole-exome sequenced). The functional significance of the Ala74Glu variant remains unknown. As mentioned in the results, TCi-HCC20-1 harbors a mutation in *Ppp1ca*, whereas TCi-HCC20-3 harbors a mutation in *Kmt2d*. *KMT2D* is one of the genes frequently disrupted by hepatitis B virus integration in human HCC (Sung et al, 2012), and mutations are commonly seen in DEN-induced HCC (Connor et al, 2018).

Although p21^+/Tert^ and p21^+/TertCi^ have distinct mutations in oncogenic drivers, they exhibit similar patterns of CNAs. For example, T-HCC662 and T-HCC663 share amplifications in Chr15 with TCi-HCC981 and TCi-HCC24, which are associated with tumor development. However, these four tumors display distinctly different transcriptional profiles, suggesting that oncogenic mutations play a major role in shaping their transcriptional landscapes. Notably, T-HCA992, TCi-HCC20-1, and TCi-HCC20-3 exhibit no or little CNA, reinforcing the idea that driver mutations primarily shape transcriptional patterns. Interestingly, half of the HCCs exhibit deletions in various genes involved in cell cycle regulation, including *Cdkn2a*, *Tp53*, and *Rb1*. Loss of these genes is likely to facilitate the bypass of cellular senescence.

Transcriptomic analysis reveals that all HCCs exhibit down-regulation of *Fbp1* and *Aldob*. This reduction has been associated with activation AKT and NRF2 pathways, along with decreased expression of p53 and p21, collectively facilitating the bypass of cellular senescence and accumulation of somatic mutations in transformed hepatocytes (Gu et al, 2023, 2025). Consistent with these findings, we observed overexpression of *Nrf2* target genes in both p21^+/Tert^ and p21^+/TertCi^ tumors. However, this up-regulation did not correlate with *Nrf2* mRNA levels, suggesting that stabilization of the Nrf2 protein, rather than increased transcription, may underlie the activation of its targets (Zhu et al, 2025). Gene expression patterns indicate the presence of a coordinated program that protects against ferroptosis, mediated by distinct gene modules. Despite some overlap, these data point to distinct strategies of ferroptosis resistance: β-catenin–driven tumors and TCi-HCC201 appear to mitigate ferroptosis by restricting labile iron and enhancing NADPH generation, whereas MAPK-activated tumors predominantly depend on an Nrf2-Slc7a11-glutathione pathway to counteract lipid peroxidation through elevated antioxidant defenses. It has recently been shown that SLC7A11 is significantly overexpressed in human HCC and that its high expression correlates with lower overall survival, thus highlighting its prognostic value (Zhang et al, 2025). Pan-cancer analysis revealed that elevated SLC7A11 associates with higher tumor mutation burden and immunosuppressive microenvironment features (Zhang et al, 2025).

Spatial characterization of tumors shows that Hnf4α levels are greatly reduced in HCC with activation of the β-catenin and hRAS pathways. Hnf4α is a central regulator of liver function and acts as a tumor suppressor in HCC (Kotulkar et al, 2024). It plays a pivotal role in hepatic glucose metabolism (Shokouhian et al, 2023) and contributes to the maintenance of hepatocyte quiescence, in part by repressing *cyclin D1* (*Ccnd1*) expression (Wu et al, 2020). Consistently, *Ccnd1* was markedly overexpressed in the tumors. In line with findings that identify Hnf4α as a key regulator of the transition from slow-growing tumors to aggressive HCC (Lazarevich et al, 2010), of note, T-HCA992 and TCi-HCA984 exhibited intermediate levels of Hnf4α expression. Surprisingly, we observed that Hnf4 alpha levels were down-regulated at the post-transcriptional level. In human colon cancer, phosphorylation of HNF4α by Src tyrosine kinase has been associated with the loss of specific isoforms of HNF4α (Chellappa et al, 2012). Degradation of HNF4α was further confirmed in the liver (Huck et al, 2019). As noted above, Src was up-regulated in all tumors, albeit to a lesser extent in T-HCC662, raising the possibility that kinases, such as Src, may promote Hnf4α degradation in these tumors. Notably, p21 levels remained elevated in the tumors, which may reflect a requirement to sustain Tert (or TertCi) expression. At the same time, increased CDK and cyclin activity could counteract p21-mediated cell cycle inhibition, allowing continued proliferation despite its presence.

We next analyzed immune cell infiltration in two distinct regions of the tumor. Despite the small size of these regions (1–2 mm^2^), the analysis revealed comparable patterns of immune infiltration across both areas of the tumors. Constitutive activation of β-catenin has been shown to suppress immune cell recruitment (Lehrich & Monga, 2025). Approximately 70% of WNT/β-catenin–active tumors are classified as immune-excluded, characterized by low T-cell infiltration (Montironi et al, 2023). It has been shown that the immune evasion associated with β-catenin activation observed in both murine and human HCC results from impaired dendritic cell recruitment (Ruiz de Galarreta et al, 2019). Consistent with these findings, T-HCC662 and T-HCC663, which exhibit high expression of *Axin2* and *Glul,* exhibited low infiltration by T lymphocytes, as well as by macrophages and dendritic cells. Chemokines known to attract T-lymphocytes (Ccl2, Ccl5, Ccl17, Ccl22, Cxcl9, Cxcl10, and Cxcl11) showed variable levels of expression across all tumors, and their levels could not explain the reduced T lymphocyte infiltration in T-HCC662 and T-HCC663. Only *Cxcl12* was consistently down-regulated in these two tumors, as well as in several other HCCs. The observed immune exclusion may therefore reflect, at least in part, tumor-specific adaptations in metabolic reprogramming and oxidative defense.

**Figure S6.**
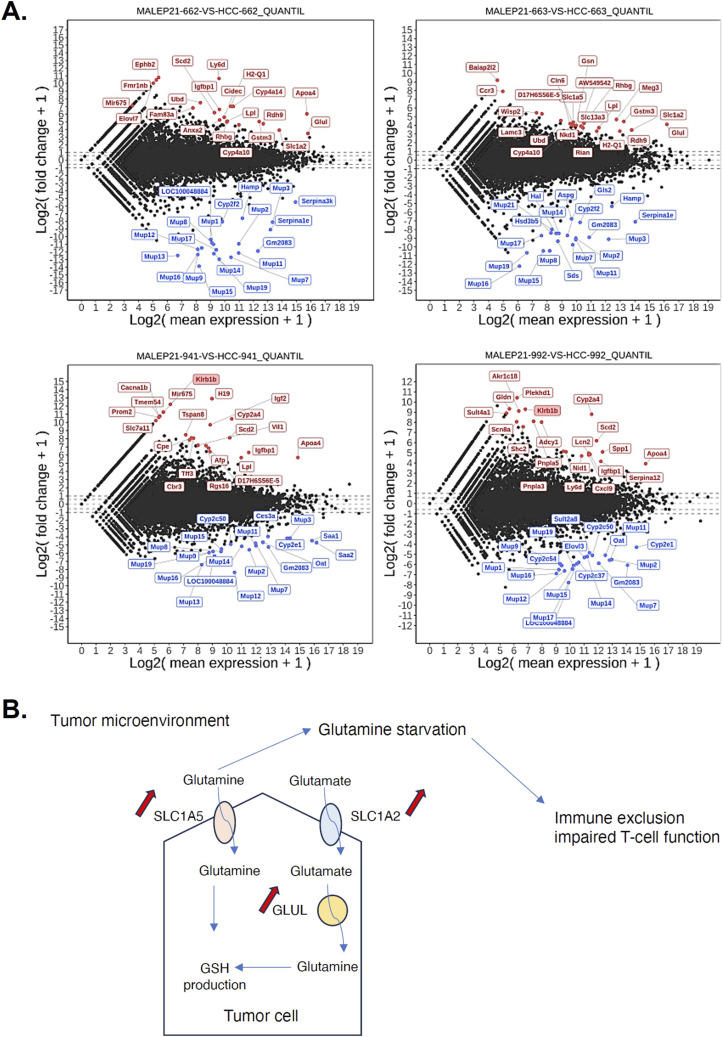
Expression changes in p21^+/Tert^ tumors. **(A)** The MA-plots (mean-average plot) highlights the genes that are most differentially expressed in the indicated tumors. **(B)** Proposed model explaining tumor immune evasion in T-HCC662 and T-HCC663. Both β-catenin–activated tumors establish a glutamine-depleted microenvironment consistent with the immune-excluded phenotype. Increased glutathione (GSH) production may also contribute to enhance antioxidant defenses.

Interestingly, Glul-overexpressing tumors T-HCC662 and T-HCC663 specifically exhibited a ∼20-fold and a ∼10-fold up-regulation of *Slc1a5*, whose gene product transports glutamine into cancer cells (Bhutia et al, 2015), and of the glutamate transporter *Slc1a2*, respectively. We predict that T-HCC662 and T-HCC663 establish a glutamine- and glutamate-depleted tumor microenvironment by combining high-affinity uptake transporters (Slc1a2 and Slc1a5) with intrinsic glutamine synthesis (GLUL), thereby achieving metabolic autonomy while starving immune and stromal cells of critical nitrogen sources. Recently, glutamine was shown to function as a metabolite regulating the cross-talk between tumors and conventional type 1 dendritic cells (cDC1s), thereby licensing cDC1s to activate cytotoxic T cells (Guo et al, 2023). It may therefore be that tumor evasion in T-HCC662 and T-HCC663 results from reduced glutamine in the TME (Fig S6). The elevated intracellular glutamine concentration may also promote increased glutathione production and strengthen antioxidant defences. Whether this principle extends to human β-catenin–activated tumors remains to be determined. Recent analyses in human HCC revealed that SLC1A5 expression was associated with poor prognosis and an immunosuppressive microenvironment, providing a marker to predict immunotherapy response (Zhang et al, 2024a, 2024b). hRAS-activated tumors on their side exhibit preferential up-regulation of SLC7A11 that imports cystine in exchange for glutamate to support antioxidant defense and protection against ferroptosis (Li et al, 2024).

## Materials and Methods

### Animals

WT (C57BL/6J N), p21^+/Tert^, and p21^+/TertCi^ mice were housed under controlled environmental conditions: temperature (21 ± 1°C), humidity (60 ± 10%), and a 12-h light/dark cycle. Animals were fed a standard diet (A04; SAFE Diet). All procedures were conducted in accordance with institutional animal care guidelines and were approved by the Institutional Ethics Committee No. 16, Paris, France (license number 16-090). Throughout the study, mice underwent regular assessments, including body weight monitoring and metabolic evaluations. Mice were euthanized at over 18 mo of age, regardless of the presence or absence of tumor-related signs. Liver tissues were collected and processed for subsequent analyses.

### Histologic characterization of tumors

Freshly collected mouse liver tissues were fixed in 10% neutral buffered formalin for 24–48 h (fixative:tissue volume ratio of 10:1), transferred to 70% ethanol for 24 h before paraffin embedding. Paraffin blocks were cut into 5-μm-thick sections, which were subsequently dried overnight at 37°C and stained with haematoxylin-eosin-saffron using an automated slide stainer before digitalization. The description and diagnosis of neoplastic and non-neoplastic mouse liver lesions followed the International Harmonization of Nomenclature and Diagnostic Criteria (INHAND) guidelines for lesions of the rat and mouse hepatobiliary system and the accepted consensual terminology used in mouse pathology, as published by international committees and experts (Deschl et al, 2001; Thoolen et al, 2010).

The researcher conducting the histological evaluation (P.C.) was aware of sample identity during the analysis. Liver neoplasms were evaluated for tumor architecture and relationship with adjacent tissue, growth pattern, areas of necrosis, neoplastic cell morphology, cytonuclear atypia, stromal composition, and leukocyte infiltration. Regional or distant metastasis, invasive growth or lympho-vascular invasion, tumor size, high mitotic activity, and severe atypia were considered key criteria of malignancy by decreasing order of importance.

### Whole exome sequencing and CNAs

Samples were sequenced to an average depth of 201× and 194× for the tumor and normal samples, respectively. Raw reads were aligned to the mouse reference genome (UCSC mm10) using BWA-MEM. Then, duplicated reads were marked with Picard Tools, and base quality scores were recalibrated using GATK (Van der Auwera et al, 2013). Somatic variant calling was performed using 12 different variant callers FreeBayes (Garrison & Marth 2012 *Preprint*), LoFreq, MuSE (Y. Fan et al, 2016), MuTect (Cibulskis et al, 2013), MuTect2 (McKenna et al, 2010), Pindel (Ye et al, 2009), Scalpel (Fang et al, 2016), Seurat (Christoforides et al, 2013), SomaticSniper (Larson et al, 2012), Strelka (Kim et al, 2018), VarDict (Z. Lai et al, 2016), and VarScan2 (Koboldt et al, 2012). A Panel of Normals (PON) was built using GATK MuTect2. An ensemble approach between the variant callers was used to filter false-positive variants. A minimum of five callers and four callers were required to retain a somatic SNV and indel, respectively. Finally, somatic variants found in the PON were filtered out.

CNAs were called using VarScan2 Copy Caller. Adjusted log2 ratios were then analyzed with circular binary segmentation as implemented in the DNA copy R/Bioconductor package (Olshen et al, 2004) to translate log2ratios measurements in regions of equal copy number. We used a threshold value of 0.1 to call the CNA alterations.

### Mutagenesis

Single-base substitutions were described based on the pyrimidines of the Watson–Crick base pairs, resulting in six major substitution types (C>A, C>G, C>T, T>A, T>C, and T>G). These were further classified across their 16 trinucleotide contexts, defining 96 distinct single-base substitution types.

### RNA sequencing and data analysis

RNA sequencing was conducted by Novogene. RNA integrity was assessed using the RNA Nano 6000 Assay Kit on a Bioanalyzer 2100 (Agilent Technologies). Total RNA was used for library construction. mRNA was isolated using poly-T oligo-attached magnetic beads and fragmented under elevated temperature in First Strand Synthesis Reaction Buffer. First-strand cDNA was synthesized using random hexamers and M-MuLV Reverse Transcriptase (RNase H-), followed by second-strand synthesis using DNA Polymerase I and RNase H. Overhangs were converted to blunt ends, 3′ ends were adenylated, and hairpin-loop adaptors were ligated. Fragments (370–420 bp) were purified using the AMPure XP system (Beckman Coulter), PCR-amplified with Phusion High-Fidelity DNA Polymerase, and assessed for quality on the Bioanalyzer 2100. Indexed libraries were clustered using the cBot Cluster Generation System and TruSeq PE Cluster Kit v3-cBot-HS (Illumina), then sequenced on the Illumina NovaSeq platform to generate 150-bp paired-end reads.

Sequencing quality was assessed using FastQC (http://www.bioinformatics.babraham.ac.uk/projects/fastqc/) and aggregated across samples with MultiQC (v1.7) (Ewels et al, 2016). Reads with a Phred quality score below 30 were filtered out to ensure high data quality.

High-quality reads were aligned to a customized mm10 mouse reference genome, including the mCherry transgene, using Subread-align (v1.6.4) (Liao et al, 2013) with default parameters. Gene-level counts were quantified with featureCounts (v1.6.4) (Liao et al, 2014).

Differential expression analysis was performed using DESeq2 (v1.26.0) (Love et al, 2014). To account for technical variability, a batch correction was applied based on sequencing run times. Genes with Benjamini–Hochberg adjusted *P* < 0.05 were considered significantly differentially expressed.

Principal component analysis was performed using the prcomp function from R’s base “stats” package on variance-stabilized transformed counts. PCA allowed visualization of sample clustering and assessment of batch effects, ensuring biological replicates were grouped together, and that batch correction successfully minimized technical variation. GO enrichment analysis was conducted with clusterProfiler (v4.6.0) (Yu et al, 2012), focusing on Biological Process (BP) and KEGG pathway categories. A padj ≤ 0.05 was used as the significance threshold, considering an appropriate gene background. Heatmaps were generated using the heatmap.2 function (v3.1.3.1), based on DESeq2-normalized expression values. Venn diagrams were produced using Python’s matplotlib venn library. Custom R scripts were used to visualize gene signatures across DESeq2-normalized counts.

Ctnnb1-activated and axin1Δ signatures were sourced from Abitbol et al (2018). The Nrf2 signature was from Mitsuishi et al (2012a).

### Imaging Mass Cytometry (IMC)

IMC was performed using the Hyperion Imaging System (Standard BioTools), a multiplexed spatial proteomics platform that enables simultaneous detection of multiple protein markers at subcellular resolution in formalin-fixed paraffin-embedded (FFPE) tissue sections. IMC combines laser ablation with cytometry by time-of-flight (CyTOF) to detect metal-conjugated antibodies, overcoming limitations associated with fluorescence-based imaging such as spectral overlap and tissue autofluorescence (Giesen et al, 2014). Mouse liver tumor FFPE sections were processed according to standard protocols, including dewaxing, rehydration, and heat-induced antigen retrieval. Sections were stained with a validated panel of 10 metal-tagged antibodies (AMK Biotech) following the steps previously described (Elaldi et al, 2021). Nuclear staining was performed using the iridium intercalator. Regions of interest (ROIs) of ∼1–2 mm^2^ were selected on the tissue based on HES-stained sections to ensure representative sampling of both tumor and surrounding liver parenchyma and to avoid necrotic or hemorrhagic areas. Scale bars (200 μm) of the uncropped images are shown in Fig S4.

Image acquisition was conducted at a spatial resolution of 1 μm using the Hyperion Imaging System. Raw data were exported as OME-TIFF files via the MCD Viewer software (Standard BioTools). For each marker of the used IMC-panel, noise and background removal was performed using CellProfiler Software to generate an automatic binarization mask used to automatically remove the noise based on a learning approach of pixel classification. All cleaned images were subsequently reviewed by an expert to ensure the absence of residual noise.

### Image analysis and quantification

Selected ROIs from denoised images of mice liver tumor sections were analyzed as follows: nuclei were segmented from the DNA1 channel using the legacy Cyto2 model, running on cellpose 4.0.6 via the napari-serialcellpose plugin (v0.2.2) (Stringer et al, 2021). Cellprofiler 4.2.8 was used for subsequent quantification of immune marker positivity and relative areas versus total DNA. Metadata were extracted from file names. Integrated intensities of cellpose-segmented nuclei were calculated for each marker. Because denoising reduced background fluorescence to near zero, any cell exhibiting a signal above background was considered positive. The percentage of marker-positive cells relative to total cells (cellpose objects) was calculated per ROI. For area-based measurement, the areas of marker signals above the same threshold were compared relative to the total DNA area per ROI. Data means were displayed as bar plots, with the individual ROI means shown as dots. Error bars represent SEM. Representative images of segmented ROIs are displayed in figure panels for transparency. Cellprofiler pipelines are available upon request.

### Telomere Shortest Length Assay (TeSLA)

TeSLA is a method for measuring the distribution of the shortest telomeres in cells and tissues. TeSLA was performed according to the protocol described by Lai et al (2017). Briefly, 50 ng of undigested genomic DNA was ligated with an equimolar mixture (50 pm each) of the six TeSLA-T oligonucleotides containing seven nucleotides of telomeric C-rich repeats at the 3′ end and 22 nucleotides of the unique sequence at the 5′ end. After overnight ligation at 35°C, genomic DNA was digested with *Cvi*AII, *Bfa*I, *Nde*I, and *Mse*I, the restriction enzymes creating either AT or TA overhangs. Digested DNA was then treated with shrimp alkaline phosphatase to remove 5′ phosphate from each DNA fragment to avoid their ligation to each other during the subsequent adapter ligation. Upon heat-inactivation of phosphatase, partially double-stranded AT and TA adapters were added (final concentration 1 μM each) and ligated to the dephosphorylated fragments of genomic DNA at 16°C overnight. After ligation of the adapters, genomic DNA was diluted to 20 pg/μl, and 2–4 μl was used in a 25 μl PCR reaction to amplify terminal fragments using primers complementary to the unique sequences at the 5′ ends of the TeSLA-T oligonucleotides and the AT/TA adapters. FailSafe polymerase mix (Epicenter) with 1× FailSafe buffer H was used to amplify G-rich telomeric sequences. Entire PCR reactions were then loaded onto the 0.85% agarose gel for separation of the amplified fragments. To visualize telomeric fragments, the DNA was transferred from the gel onto the nylon membrane by Southern blotting procedure and hybridized with the ^32^P-labeled (CCCTAA)_3_ probe. The sizes of the telomeric fragments were quantified using TeSLA Quant software (Lai et al, 2017).

### Ki67 and gH2AX

Fresh liver was fixed in 10% phosphate-buffered formalin overnight. Paraffin wax sections of 5 μm were prepared for immunostaining (Ki67-ab15580; Abcam, gH2AX-05-636; Merck Millipore).

## Supplementary Material

Reviewer comments

## Data Availability

RNA-seq data are currently being deposited in the Gene Expression Omnibus (GSE) (GSE317255).
