# Supplementary figures and images for: TERT drives liver tumorigenesis beyond telomere elongation
Source: Life Sci Alliance. 2026 Jul 2;9(9):e202603660. doi: 10.26508/lsa.202603660 (PMC13329133; doi:10.26508/lsa.202603660)

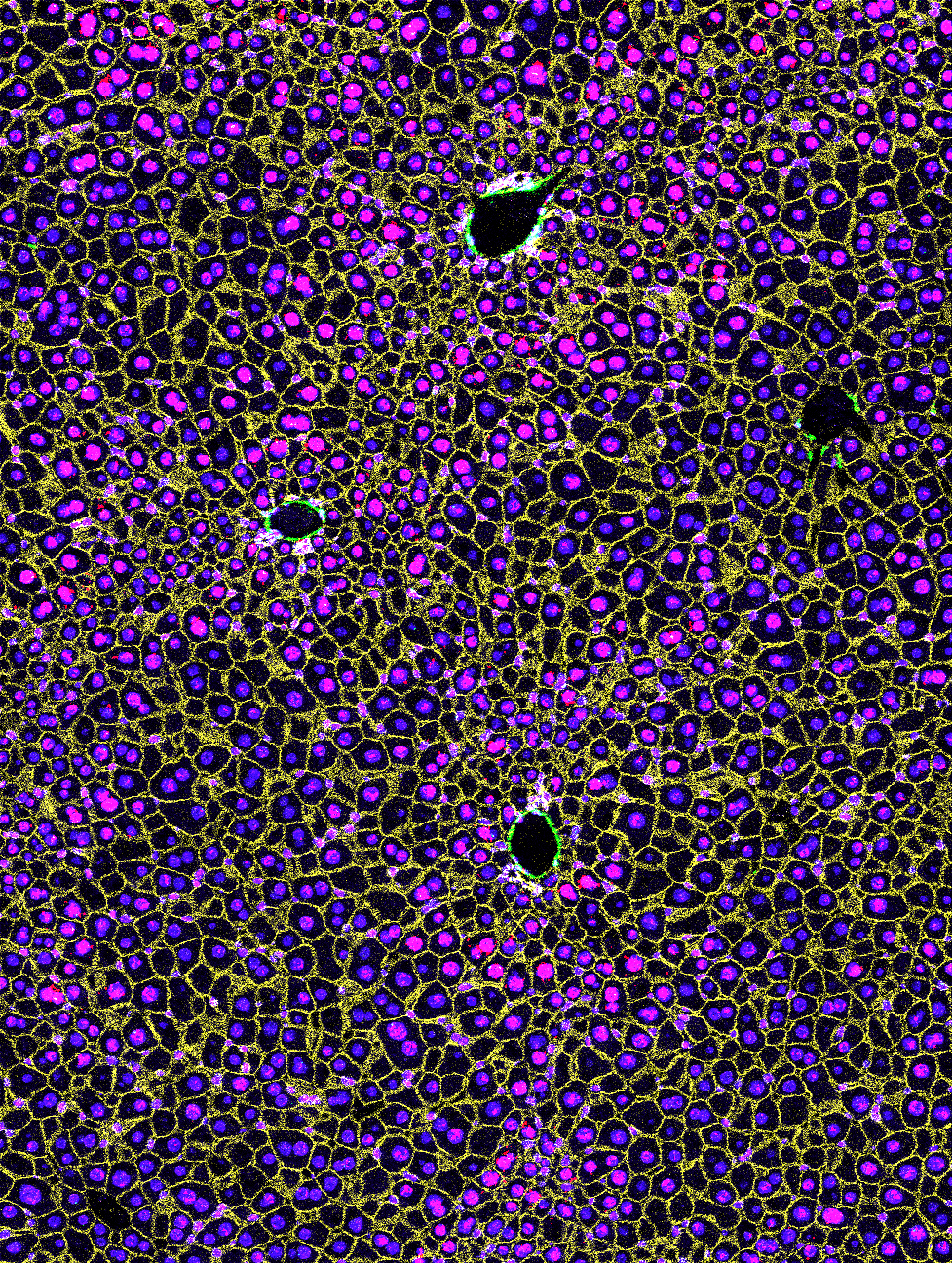

Supplement: Supplementary file 6 [file LSA-2026-03660_SdataF6.1.png]

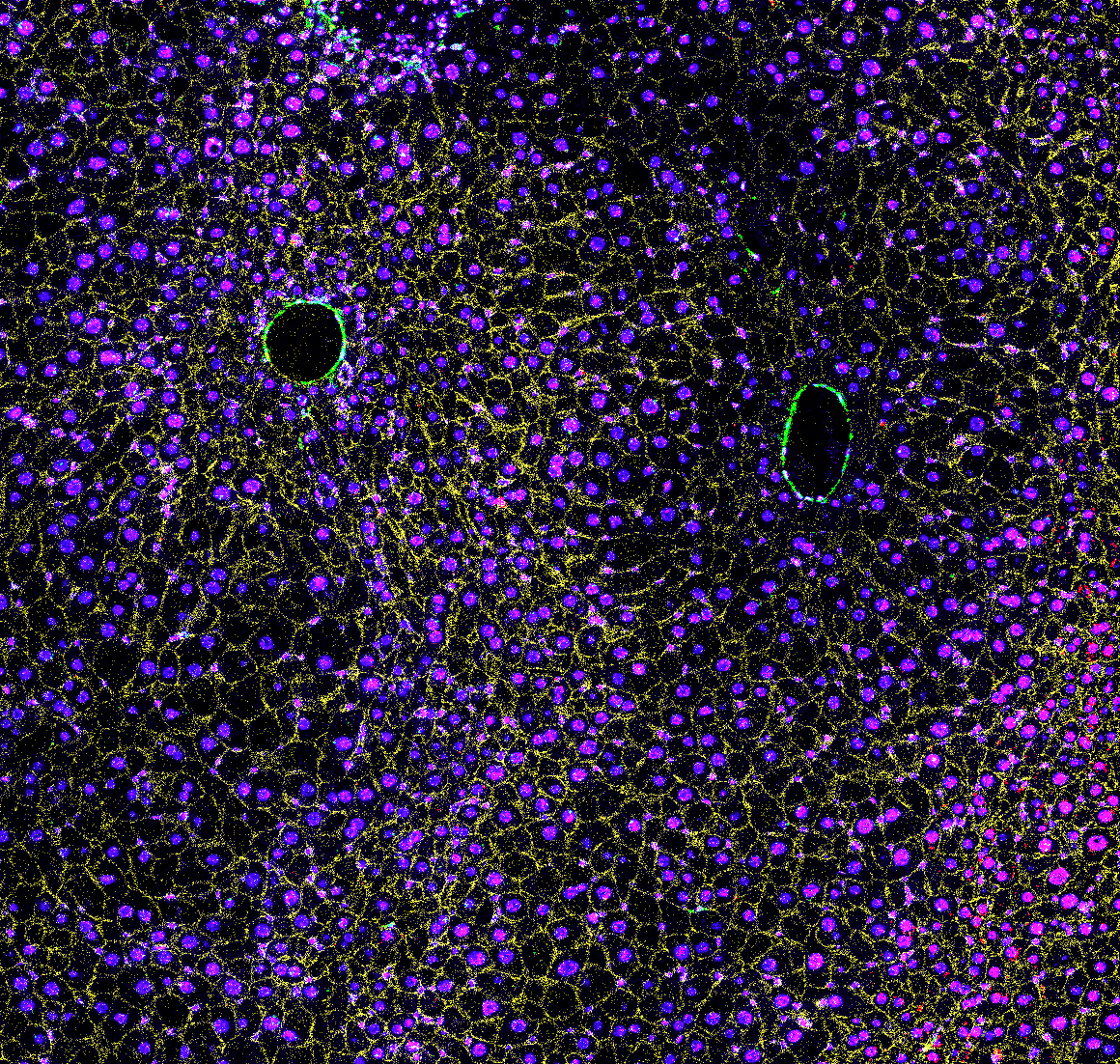

Supplement: Supplementary file 7 [file LSA-2026-03660_SdataF6.2.png]

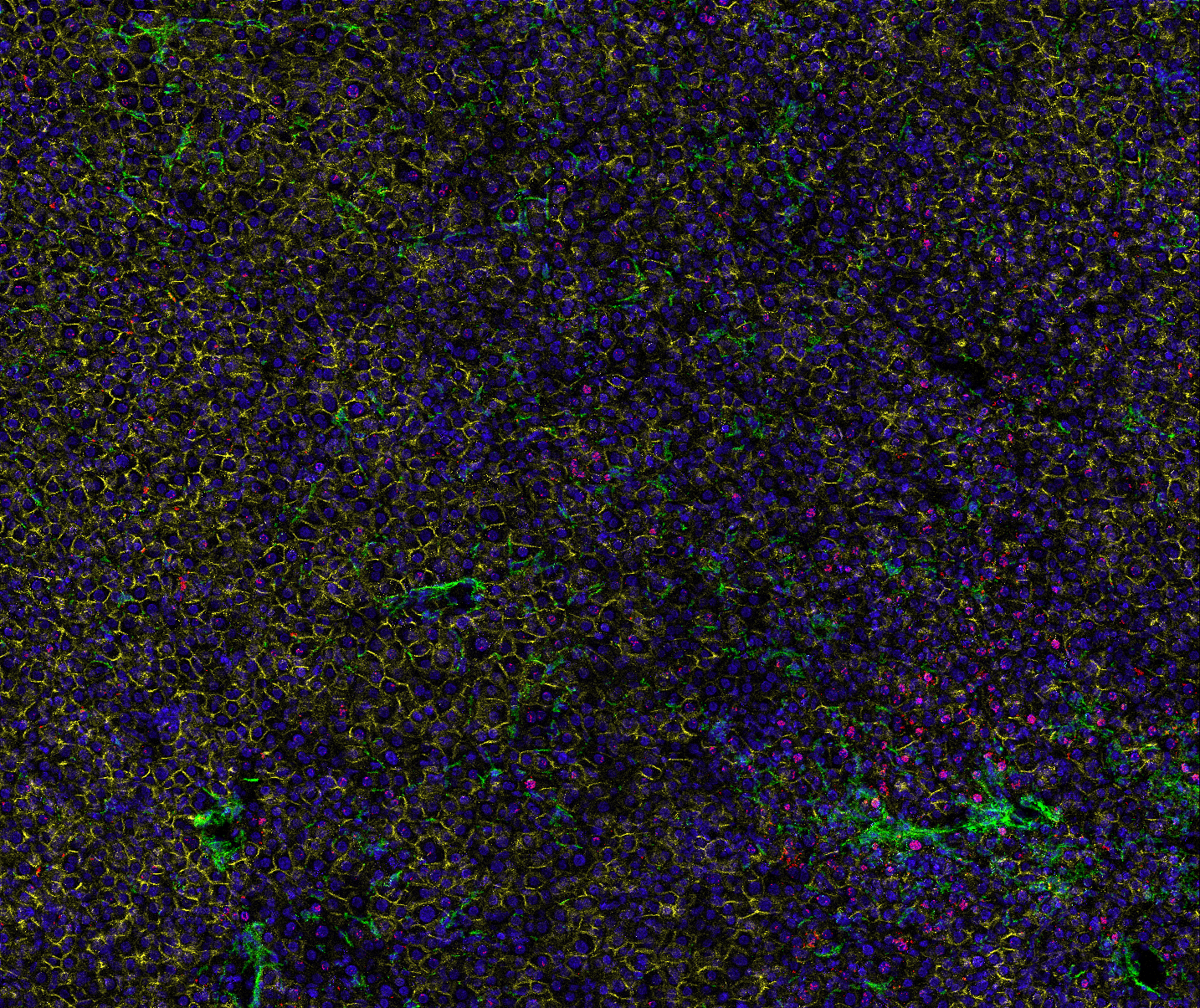

Supplement: Supplementary file 8 [file LSA-2026-03660_SdataF6.3.png]

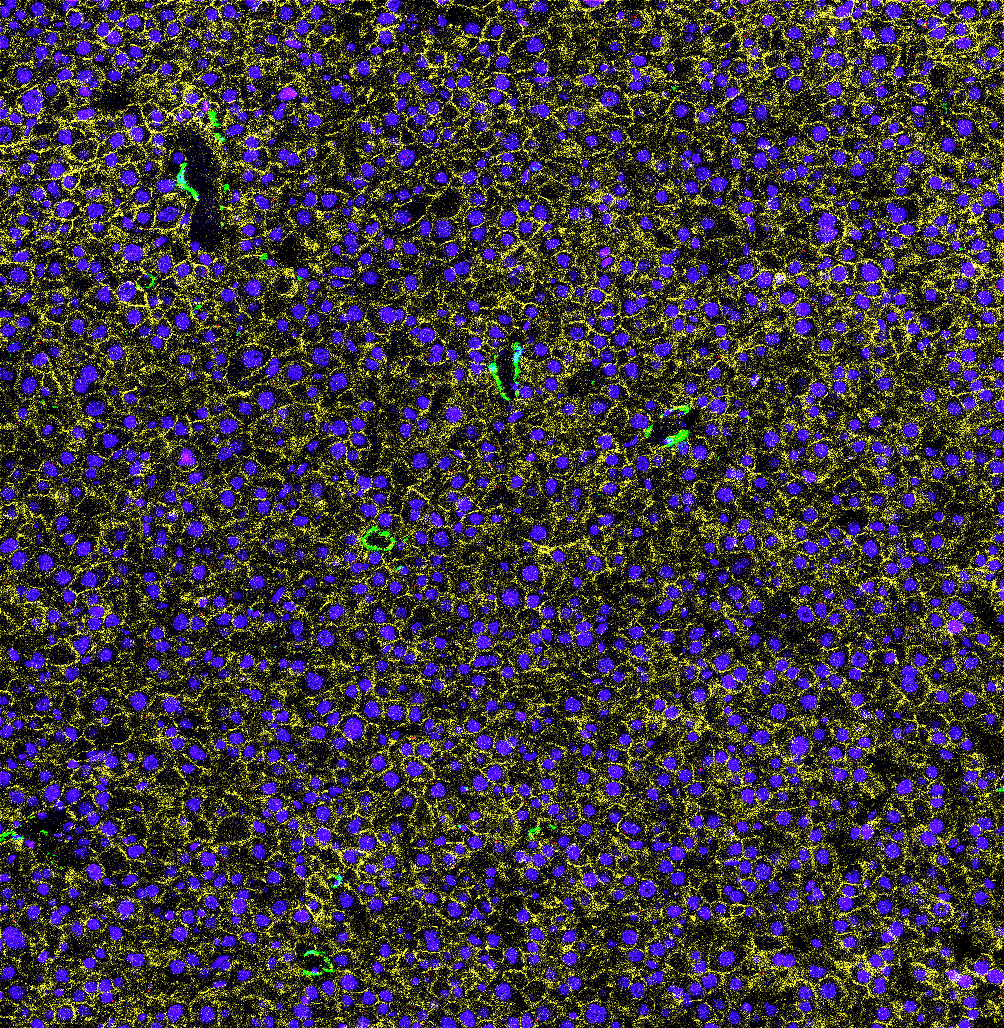

Supplement: Supplementary file 9 [file LSA-2026-03660_SdataF6.4.png]

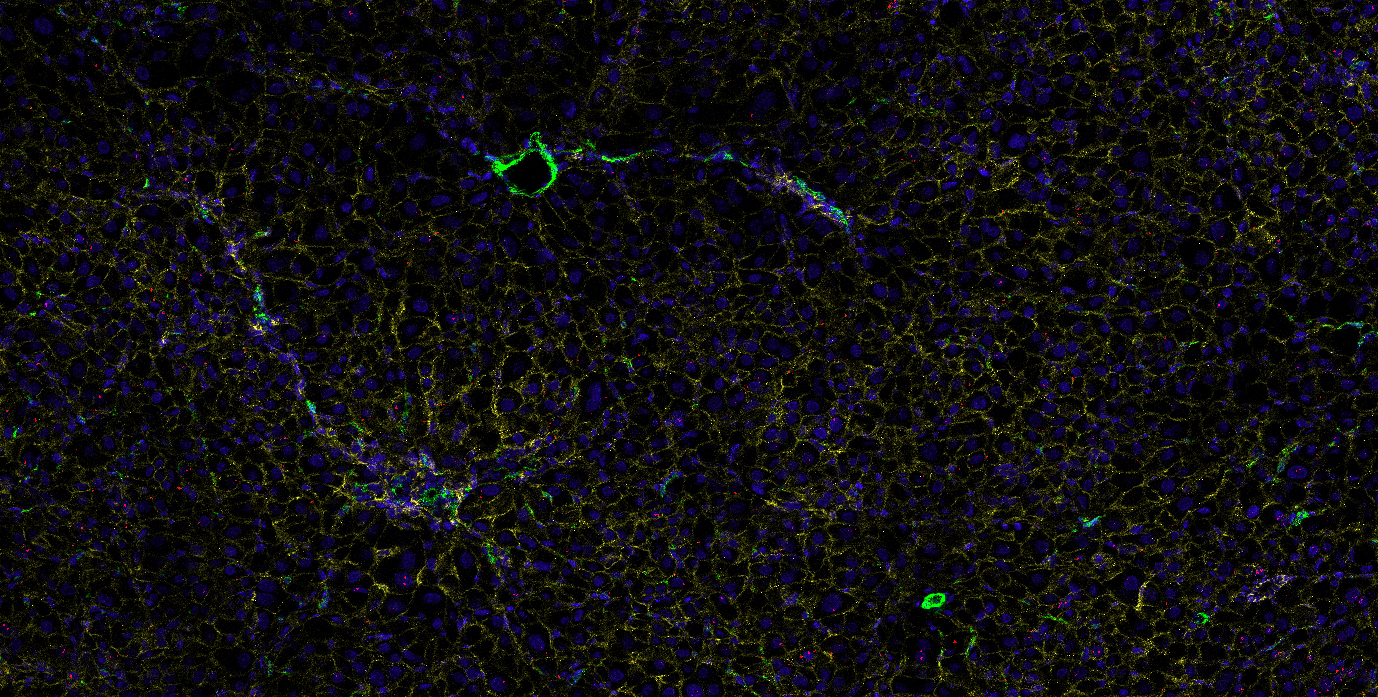

Supplement: Supplementary file 10 [file LSA-2026-03660_SdataF6.5.png]

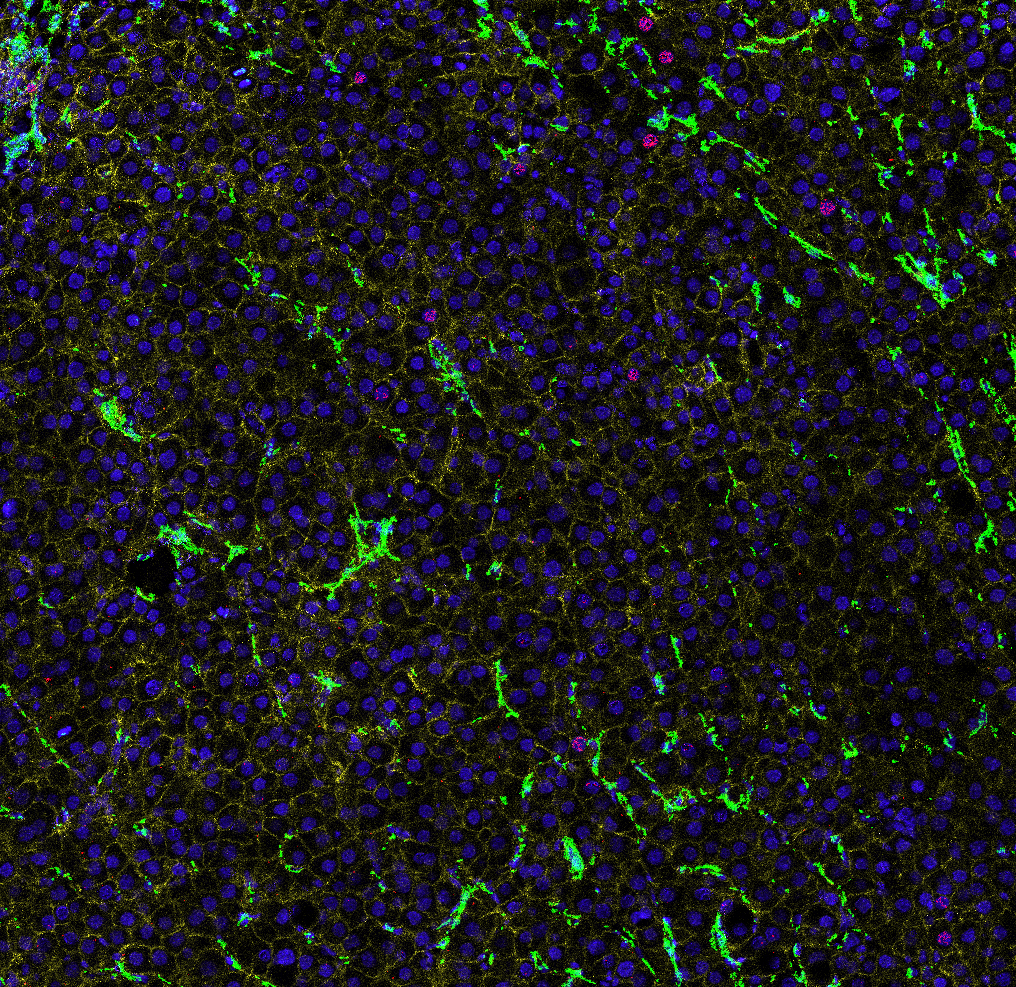

Supplement: Supplementary file 11 [file LSA-2026-03660_SdataF6.6.png]

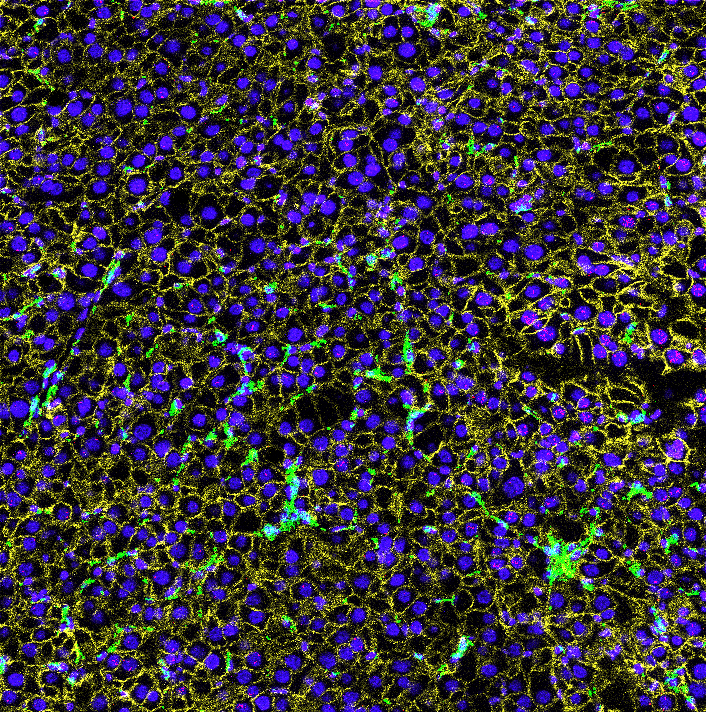

Supplement: Supplementary file 12 [file LSA-2026-03660_SdataF6.7.png]

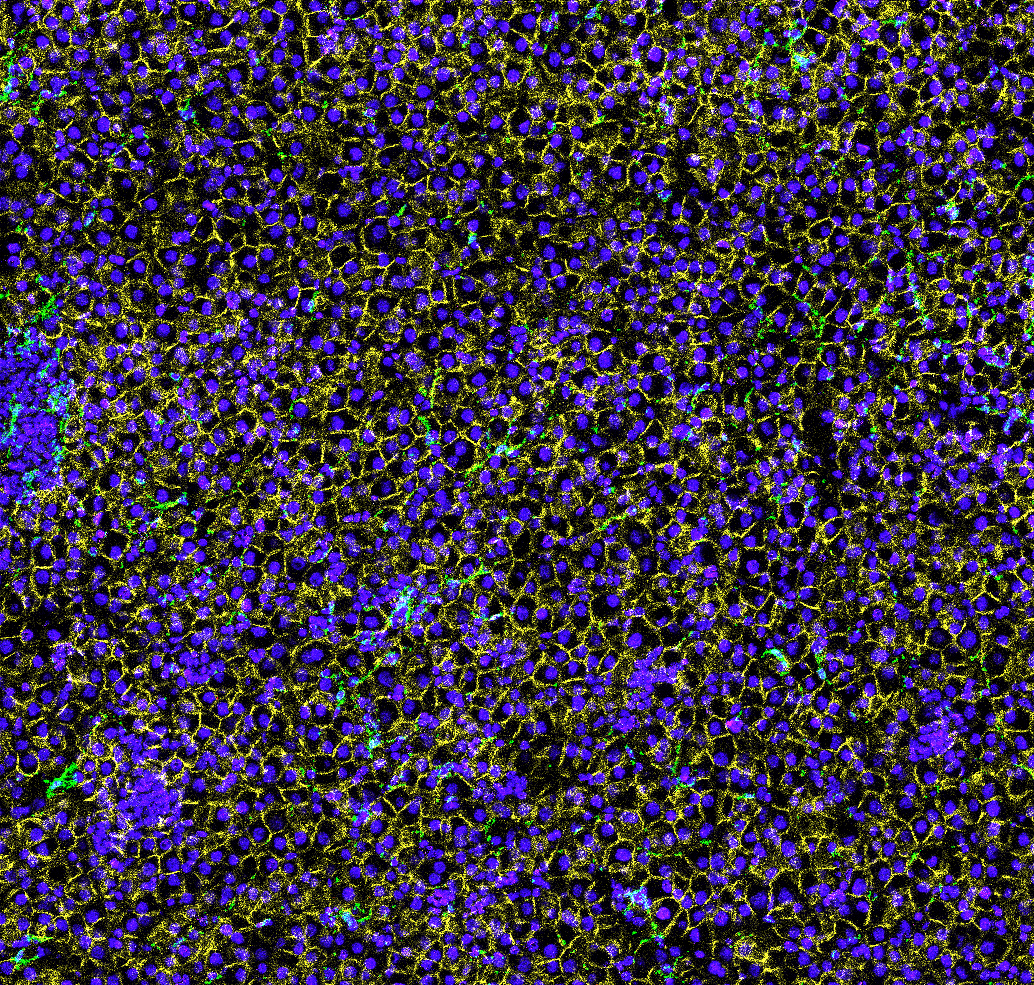

Supplement: Supplementary file 13 [file LSA-2026-03660_SdataF6.8.png]

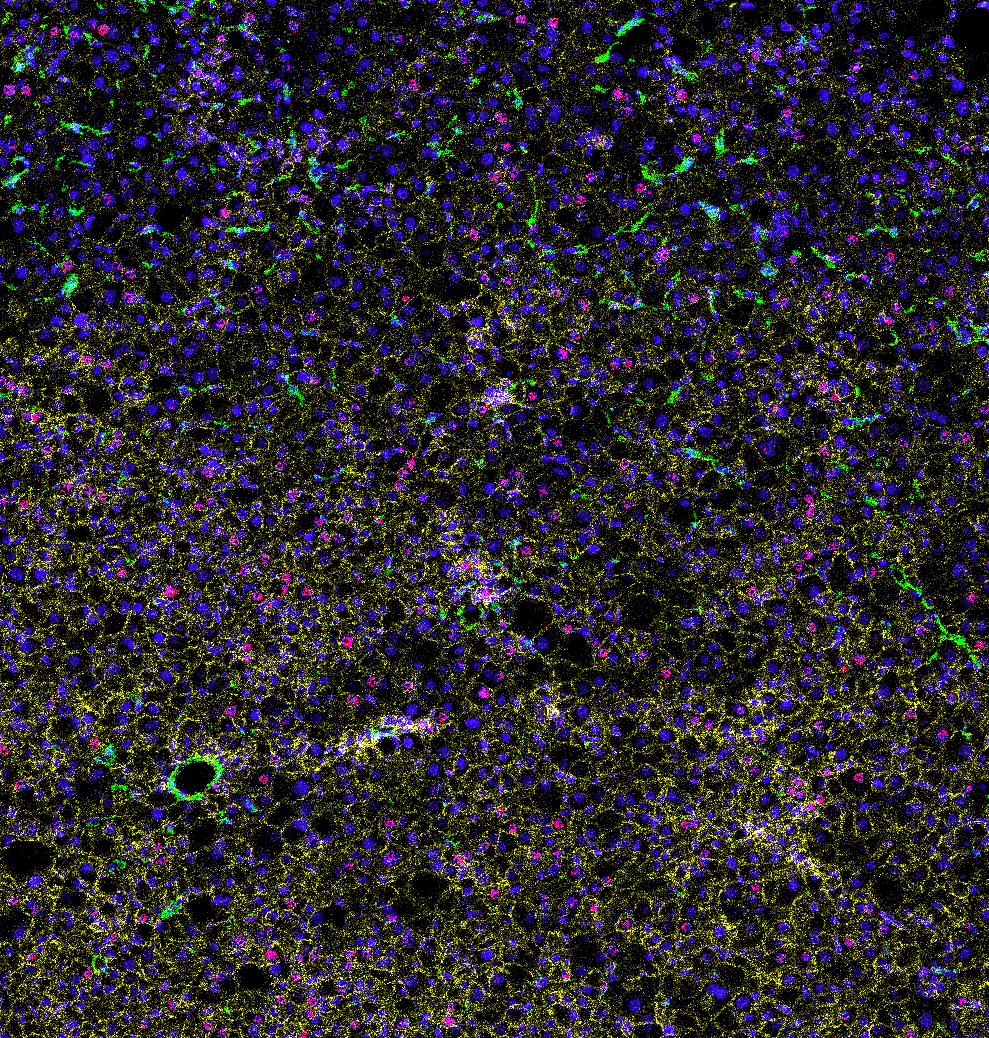

Supplement: Supplementary file 14 [file LSA-2026-03660_SdataF6.9.png]

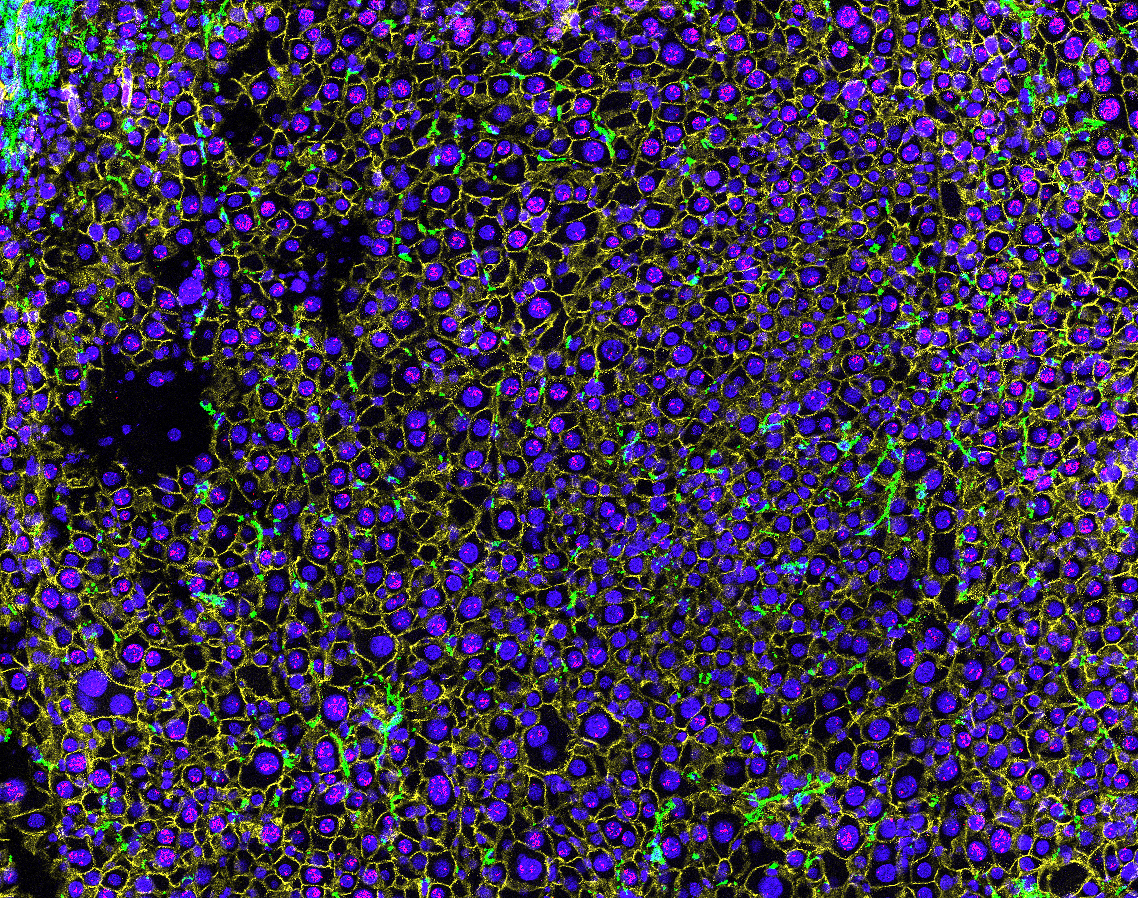

Supplement: Supplementary file 15 [file LSA-2026-03660_SdataF6.10.png]

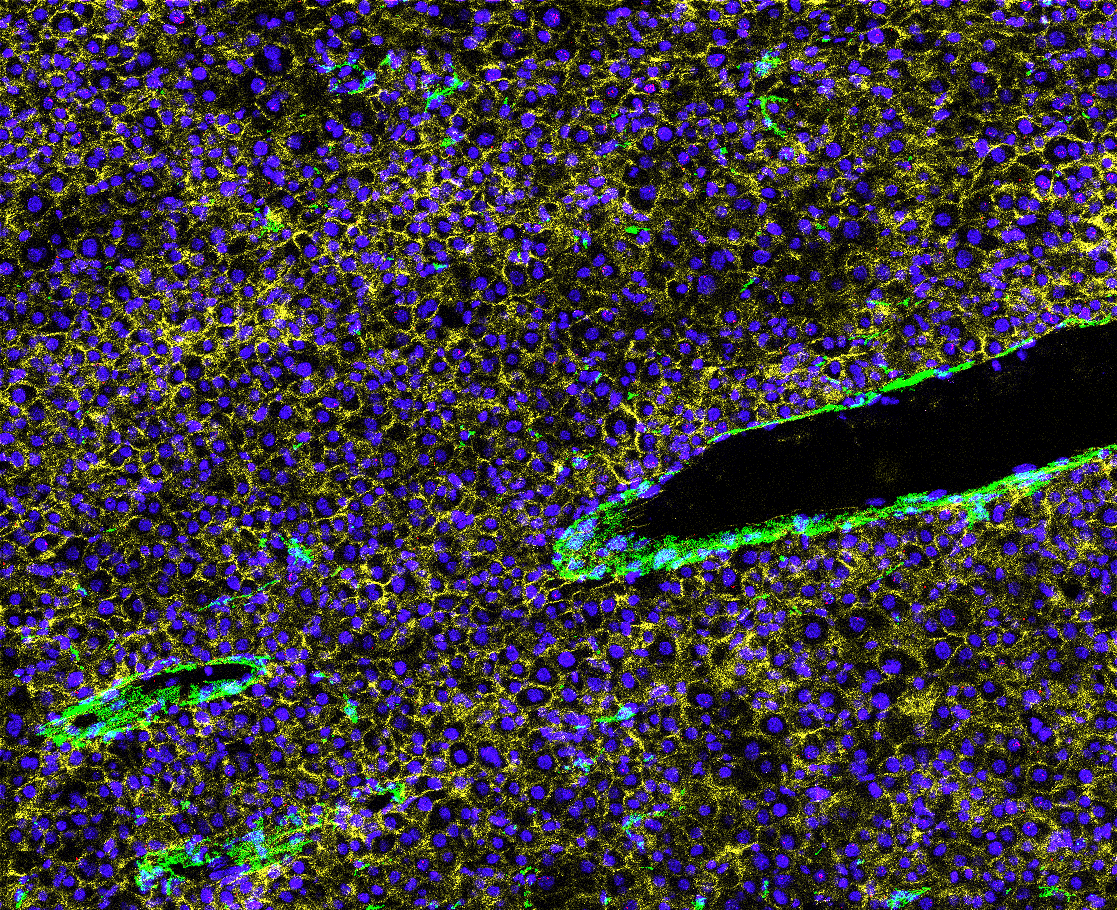

Supplement: Supplementary file 16 [file LSA-2026-03660_SdataF6.11.png]

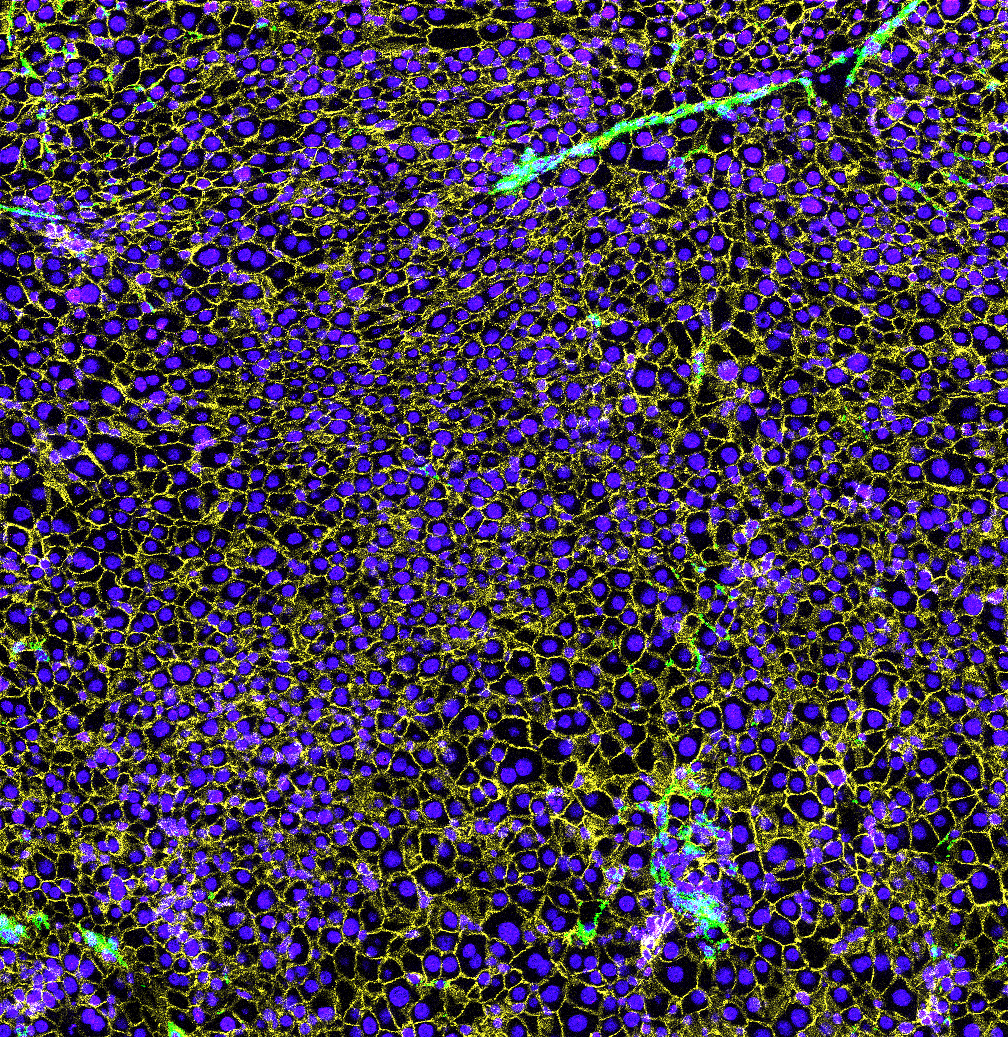

Supplement: Supplementary file 17 [file LSA-2026-03660_SdataF6.12.png]
