# Supplementary material for: TERT drives liver tumorigenesis beyond telomere elongation
Source: Life Sci Alliance. 2026 Jul 2;9(9):e202603660. doi: 10.26508/lsa.202603660 (PMC13329133; doi:10.26508/lsa.202603660)
Supplement: Reviewer comments [file LSA-2026-03660_review_history.pdf]

TERT drives liver tumorigenesis beyond telomere elongation

Laura Braud, Julien Vernerey, Arnaud GUILLE, Pierre Cordier, Clémence Ginet, Tom Egger, Manuel Bernabe, Dmitri Churikov, Quentin Da-Costa, Aida Meghraoui, Chantal Desdouets, Li Gu, François Bertucci, Christophe Lachaud, and Vincent Géli
DOI: https://doi.org/10.26508/lsa.202603660

Corresponding author(s): Vincent Géli, Centre National de la Recherche Scientifique and Laura Braud, Marseille Cancer Research Centre (CRCM)

Review Timeline:

Submission Date:	2026-02-10
Editorial Decision:	2026-03-16
Revision Received:	2026-05-05
Editorial Decision:	2026-06-01
Revision Received:	2026-06-08
Accepted:	2026-06-11

Scientific Editor: Tim Fessenden

Transaction Report:

(Note: With the exception of the correction of typographical or spelling errors that could be a source of ambiguity, letters and reports are not edited. The original formatting of letters and referee reports may not be reflected in this compilation.)

March 16, 2026

Re: Life Science Alliance manuscript #LSA-2026-03660-T

Dr. Vincent Geli
Centre National de la Recherche Scientifique
IRCAN
Nice, BDR 13273
FRANCE

Dear Dr. Geli,

Thank you for submitting your manuscript entitled "TERT drives liver tumorigenesis beyond telomere elongation" to Life Science Alliance. The manuscript was assessed by expert reviewers, whose comments are appended to this letter. We invite you to submit a revised manuscript addressing the Reviewer comments.

As you will see, reviewers were unanimous in their appreciation for these novel findings on TERT and hepatocellular carcinoma. Reviewers 1 and 3 requested a comparison of telomere lengths in tumors from the two mouse models. Reviewer 2 sought clarification on clustering analyses shown in Figures 4 and 5, and clearer support for claims on glycolysis and NRF2 pathway signaling. We concur these new analyses should be provided or else claims adjusted to more closely align with the observations. Finally we bring your attention to LSA policy that prohibits statements relying on unpublished results (Line 446).

I would be happy to discuss the revision in more detail via email or phone/videoconferencing if you wish. Please let me know which option you prefer, if any.

To upload the revised version of your manuscript, please log in to your account: https://lsa.msubmit.net/cgi-bin/main.plex

You will be guided to complete the submission of your revised manuscript and to fill in all necessary information. Please get in touch in case you do not know or remember your login name.

The typical timeframe for revisions is three months. Please note that papers are generally considered through only one revision cycle, so strong support from the referees on the revised version is needed for acceptance.

While you are revising your manuscript, please also attend to the below editorial points to help expedite the publication of your manuscript. Please direct any editorial questions to the journal office. When submitting the revision, please include a letter addressing the reviewers' comments point by point.

Thank you for this interesting contribution to Life Science Alliance. We hope that the comments below will prove constructive as your work progresses, and we are looking forward to receiving your revised manuscript.

Sincerely,

Tim Fessenden
Scientific Editor
Life Science Alliance

A. THESE ITEMS ARE REQUIRED FOR REVISIONS

-- A letter addressing the reviewers' comments point by point.

-- An editable version of the final text (.DOC or .DOCX) is needed for copyediting (no PDFs).

-- High-resolution figure, supplementary figure and video files uploaded as individual files: See our detailed guidelines for preparing your production-ready images, https://www.life-science-alliance.org/authors

-- Summary blurb (enter in submission system): A short text summarizing in a single sentence the study (max. 200 characters including spaces). This text is used in conjunction with the titles of papers, hence should be informative and complementary to the title and running title. It should describe the context and significance of the findings for a general readership; it should be

written in the present tense and refer to the work in the third person. Author names should not be mentioned.

-- By submitting a revision, you attest that you are aware of our payment policies found here: https://www.life-science-alliance.org/copyright-license-fee

B. MANUSCRIPT ORGANIZATION AND FORMATTING:

Full guidelines are available on our Instructions for Authors page, https://www.life-science-alliance.org/authors

We encourage our authors to provide original source data, particularly uncropped/-processed electrophoretic blots and spreadsheets for the main figures of the manuscript. If you would like to add source data, we would welcome one PDF/Excel-file per figure for this information. These files will be linked as supplementary "Source Data" files.

IMPORTANT: It is Life Science Alliance policy that if requested, original data images must be made available. Failure to provide original images upon request will result in unavoidable delays in publication. Please ensure that you have access to all original microscopy and blot data images before submitting your revision.

Reviewer #1 (Comments to the Authors (Required)):

Braud et al. engineered two KI mouse models that express either the catalytic subunit of telomerase (Tert) or a catalytically inactive mutant (TertCi) under the control of the p21 promoter. In both models, called $p21^+$/Tert and $p21^+$/TertCi, they observed the arising of hepatocellular carcinomas (HCC) or hepatocellular adenoma (HCA) in mice older than 18 months. The tumors display multiple features that closely resemble those observed in human HCC. $p21^+$/Tert tumors harbored activating mutations in Ctnnb1 associated to Kdm mutations, in Egfr, or in subunits of the phosphatase Ppp1, whereas $p21^+$/TertCI tumors predominantly carried activating mutations in either Hras or Ep300. The tumors arising in both models displayed copy number alterations that are frequently observed in human HCC. They show that tumors from the $p21^+$/Tert and $p21^+$/TertCi mice show a higher frequency of C>A and C>T mutations. They further analyzed the transcriptomic profiles, which were consistent with the specific oncogenic mutations identified in each tumor. They demonstrate that HCCs from both genotypes undergo metabolic reprogramming, highlighted by downregulation of FBP1, a change previously associated with activation of NRF2 signaling. However, $p21^+$/Tert and $p21^+$/TertCI tumors show selective overexpression of different sets of NRF2 target genes. Finally, they employed the Hyperion{trade mark, serif} Imaging System to profile multiple markers in FFPE tumor samples. Their findings reveal that HNF4α levels are markedly reduced post-transcriptionally, accompanied by a widespread distribution of α-SMA-positive cancer-associated fibroblasts particularly in HCC with activation of the β-catenin and hRAS pathways. They further quantified liver-infiltrating lymphocyte, macrophage, and dendritic cell subsets, revealing that two β-catenin-activated tumors had the lowest immune cell infiltration.

This study introduces two novel HCC mouse models that spontaneously develop tumors in aged mice without any external induction. The authors note in the discussion that these mice exhibit hepatic steatosis by 9 months of age, indicating that these models may recapitulate the progression from MASH to HCC. Furthermore, these models recapitulate TERT reactivation in presenescent hepatocytes during the early phases of tumorigenesis, occurring in tumors with intact wild-type p53.

This is an original and important study that reveals that the function of TERT in HCC development surpasses its traditional role in telomere elongation. Furthermore, it uncovers new insights into the potential role of phosphatases in tumorigenesis, mechanisms by which tumors defend against oxidative stress and ferroptosis, and the regulation of Hnf4α. The discussion is comprehensive and insightful, thoughtfully proposing a mutagenic role for TERT and highlighting glutamine and glutamate transporters as promising targets for future research.

The study would benefit from additional details:

1) Are the telomeres in p21+/TertCi tumors shorter than those in p21+/Tert tumors?

2) Could the authors indicate the positions of the regions of interest (ROIs) within the tumors?

Reviewer #2 (Comments to the Authors (Required)):

Summary :
Braud et al. explore the implication of non-canonical signaling pathway of TERT expression in liver carcinogenesis, they generated two mouse models under p21 promoter , one expresses an active form of TERT while the other expresses an inactive form. Both models develop liver tumors at 18-20 months old mice. The study is descriptive of those tumors.
The model implies the expression of TERT in dormant cells (G0/G1) as P21 is expressed in these cells. When TERT is expressed it can degrade p21 as it is demonstrated in the literature, leading to a proliferative state. We can therefore understand the development of tumors in the TERT-WT model, interestingly, they find tumors in the TERT-inactive model as well, which suggest an oncogenic activity of TERT beyond telomere maintenance. Nevertheless, the presence of tumors in the control group mitigates the results as we are not aware of the number of animals used for the whole study, as well as what other factors might generate tumors especially in old mice.
The main statement of the article is new and valid but any further interpretation is compromised as the authors do not go further in validating the WES and RNAseq data. The authors remain with a descriptive and literature validation approach.
WES, RNAseq data give indications to potential targets, for example Figure 5 suggests that glycolysis and NRF2 are differentially expressed in the tumors, we need further validation at protein or mRNA level.
Even if further work is needed, the study is of interest to the field of genetics of liver cancer. It suggests a new role of TERT. The model is ingenious and interesting to explore the implication of dormant cells in cancer development.

Major comments :
1. How did you check that the TERT_Ci is really inactive ? As from the WES and RNAseq, we don't see differences in TERT expression.
2. Table1 , the wild-type mice develops liver hyperplasia and hemangiosarcoma, how many control mice were used, how do you differentiate between the spontaneous disease and the induced malignancy due to the vector
3. Fig 4 and Fig5, the clustering of the tumors is not demonstrated, we see a partial clustering of the genes that are supposed to form signatures, but the tumors are not clustered. Another analysis of the data should be performed to determine if the different tumors cluster by group.
4. Authors state that in figure 5A, all tumors overexpress glycolytic enzymes, this can not be deduced from the figure (group 2 genes are mostly blue = downregulated)
5. Here the authors state that LT662 and LT663, Fig5C, express group 1 genes, we cannot view the group on the graph, therefore we can not come to the same conclusion
6. The conclusions on Glycolysis and NRF2 role are not solid as displayed. I suggest a better presentation of the results with a clustering of the tumors to come to a signature associated with each tumor model.
7. As the model relies on p21 expression, did you check for senescence ?

Minor comments :
8. Line 157, there is an error displayed
9. Linz 245, "et" written is French
10. Line 246 to Line 253, wrong referencing of the panels in the figure 3. 3C should be 3D and 3D should be 3E
11. Respect the nomenclature for gene names and protein names should be observed.
12. The categories of tumors (WT, TERT or TERT-Ci) should be recalled in the figures to facilitate their interpretation, in addition of the acronym HCC, HCA or HP for each tumor. For example, LT662_HCC or HCC662.
13. To decrease the number of figures, the microscopy images can be reduced to one representative image per type of tumor or category of interest. The rest should appear in the supplementary.
14. Figure 8 and 9 should be merged together.
15. Table S1 does not display all tumors or tissues describe in the paper (L1069-2, L982-2), in relation to this, try to show always the same tumors in all figures, there are some where we only see a fraction without justification.
16. The discussion is lengthy and should highlight only important points.

Reviewer #3 (Comments to the Authors (Required)):

The authors of this article have used two mice models previously described that express the TERT gene coding region under control of the p21 promoter. One of the models express the wild-type TERT protein while the other express one catalytically inactive variant of this protein. The authors report that mice from both models develop liver tumors by 18-20 months of age. They conclude from this observation that TERT expression drives liver tumorigenesis independently of the catalytic activity of the enzyme and, therefore, of their telomere functions. In addition, the authors characterize extensively the liver tumors generated showing that they present histopathological features resembling human hepatocellular carcinomas (HCCs). The presence of driver mutations in these tumors, their transcriptome profile, signaling pathways activation and immune cells infiltration are also carefully analyzed.
One interesting observation is that different driver mutations are found in the two mice models. Activating mutations in the Ctnnb1 gene, coding for -catenin and in the genes coding for PP1 (protein phosphatase 1) subunits were observed in the

tumors over-expressing wild type TERT. In contrast, the activating Hras mutation (HrasGln61Lys) was observed in several tumors over-expressing the mutant TertCi protein. These tumors also carried mutations in PP1 subunits and three of them the Ep300Ala74Glu variant. Tumors from both mice models showed increased glycolysis, suppression of gluconeogenesis, reduced HNF-4 -positive hepatocytes and diminished immune cells infiltration. The authors conclude that "these findings uncover telomere-independent functions of TERT and identify molecular and metabolic features with potential relevance for predicting immunotherapy response".

The data reported in this article are extensive and very sound. Even so, there are some questions that still remain open about the role of TERT and the inactive mutant variant, on liver tumorigenesis. Current models propose that tumor development require some mechanism of telomere elongation being induced. Many tumors activate TERT expression to induce telomere elongation. This mechanism could be mimicked upon TERT expression under the control of p21 promoter in senescent cells, as discussed by the authors. In the case of the expression of the TERT mutant this mechanism of direct telomere elongation is discarded. One possible mechanism of elongation could be over-expression of the endogenous TERT gene but the data presented in the article rather indicated a decrease in TERT expression. It would be very interesting to determine the size of the telomeres in the tumors present in the mice that overexpressed the TERT mutant and to compare it with that of the tumors induced by wild-type TERT. In the case that telomere elongation is observed after mutant TERT expression is would be very interesting to consider the mechanisms involved given that the expression of endogenous TERT is not induced. Could the alternative mechanism of telomere elongation (ALT) be induced? Telomere length analysis in the tumors in the two mice models could provide some information about these possible alternatives.

A second mechanistic question still open is the reason by overexpression of TERT or the mutant protein induce different tumorigenic pathways. Could these two protein variants interact with different molecular targets? Could be these differences due to the relatively small number of tumors analyzed? These and other possible alternatives could be considered in more detail in the Discussion section.

Minor points:

1. The proportion of mice that develop liver tumors is estimated in 25% in the Abstract section (line 34) and 15% in the Introduction (line 88)
2. There is a typographic mistake in line 157
3. Line 206, "reported approx." might be "reported in approx."
4. There is a typographic mistake in line 245 "35% et 45%)
5. Line 663. The statement "was not blinded" should be revised.

Point-by-point response to reviewers Life Science Alliance

Reviewer #1

Braud et al. engineered two KI mouse models that express either the catalytic subunit of telomerase (Tert) or a catalytically inactive mutant (TertCi) under the control of the p21 promoter. In both models, called p21^{+}/Tert and p21^{+}/TertCi, they observed the arising of hepatocellular carcinomas (HCC) or hepatocellular adenoma (HCA) in mice older than 18 months. The tumors display multiple features that closely resemble those observed in human HCC. p21^{+}/Tert tumors harbored activating mutations in Ctnnb1 associated to Kdm mutations, in Egfr, or in subunits of the phosphatase Ppp1, whereas p21^{+}/TertCI tumors predominantly carried activating mutations in either Hras or Ep300. The tumors arising in both models displayed copy number alterations that are frequently observed in human HCC. They show that tumors from the p21^{+}/Tert and p21^{+}/TertCi mice show a higher frequency of C>A and C>T mutations. They further analyzed the transcriptomic profiles, which were consistent with the specific oncogenic mutations identified in each tumor. They demonstrate that HCCs from both genotypes undergo metabolic reprogramming, highlighted by downregulation of FBP1, a change previously associated with activation of NRF2 signaling. However, p21^{+}/Tert and p21^{+}/TertCI tumors show selective overexpression of different sets of NRF2 target genes. Finally, they employed the Hyperion{trade mark, serif} Imaging System to profile multiple markers in FFPE tumor samples. Their findings reveal that HNF4α levels are markedly reduced post-transcriptionally, accompanied by a widespread distribution of α-SMA-positive cancer-associated fibroblasts particularly in HCC with activation of the β-catenin and hRAS pathways. They further quantified liver-infiltrating lymphocyte, macrophage, and dendritic cell subsets, revealing that two β-catenin-activated tumors had the lowest immune cell infiltration. This study introduces two novel HCC mouse models that spontaneously develop tumors in aged mice without any external induction. The authors note in the discussion that these mice exhibit hepatic steatosis by 9 months of age, indicating that these models may recapitulate the progression from MASH to HCC. Furthermore, these models recapitulate TERT reactivation in pre-senescent hepatocytes during the early phases of tumorigenesis, occurring in tumors with intact wild-type p53. This is an original and important study that reveals that the function of TERT in HCC development surpasses its traditional role in telomere elongation. Furthermore, it uncovers new insights into the potential role of phosphatases in tumorigenesis, mechanisms by which tumors defend against oxidative stress and ferroptosis, and the regulation of Hnf4α. The discussion is comprehensive and insightful, thoughtfully proposing a mutagenic role for TERT and highlighting glutamine and glutamate transporters as promising targets for future research.

We thank referee 1 for his/her comments.

The study would benefit from additional details:

1) Are the telomeres in p21+/TertCi tumors shorter than those in p21+/Tert tumors?

The TertCi allele carries a well-characterized point mutation (D702A) that abolishes telomerase catalytic activity (Chang et al. 2008, PloS Genetics). We have previously demonstrated the loss of catalytic function of TertCi across multiple cellular contexts, including lung and smooth muscle cells (Lipskaia et al., 2024), as well as adipocytes and stem cells (Braud et al., 2025). In all systems examined, p21-driven Tert expression reduces the burden of very short telomeres, whereas p21$^{+/TertCi}$ mice do not exhibit this effect. These findings suggest that p21-driven mTert expression in aged mice can partially mitigate the accumulation of critically short telomeres in vivo, in contrast to catalytically inactive mTERT. Importantly, average telomere length remains comparable across all genotypes (the mice retain their endogeneous *Tert* locus).

In the revised manuscript, we performed TeSLA analyses on two p21$^{+/Tert}$ tumors (HCC663 and HCA992) and two p21$^{+/TertCi}$ tumors (HCC981 and HCA984), alongside corresponding non-tumoral samples. The fraction of telomeres shorter than 1 kb was comparable between control and tumor samples in p21$^{+/Tert}$ livers, whereas p21$^{+/TertCi}$ tumors exhibited an increased proportion of telomeres

below 1 kb, suggesting that $Tert^{Ci}$ is devoid of catalytic activity. TeSLA method has been added in the Material and Methods section **Line 707** and in Result section **Line 151**.

These results are consistent with additional in vivo observations. In both lung and adipose tissue of obese mice, $p21^{+/Tert}$, but not $p21^{+/TertCi}$, animals exhibit reduced oxidative damage. Furthermore, in adipose tissue, Tert expression decreases apoptotic cell abundance, whereas $Tert^{Ci}$ has no detectable effect. Collectively, these data indicate that Tert and $Tert^{Ci}$ behave distinctly in vivo and confirm that $Tert^{Ci}$, as expected from disruption of its catalytic triad, lacks telomerase enzymatic activity.

2) Could the authors indicate the positions of the regions of interest (ROIs) within the tumors?
The regions of interest were defined by pathologists based on H&E-stained tissue sections. ROI are shown below in response to Reviewer 1.

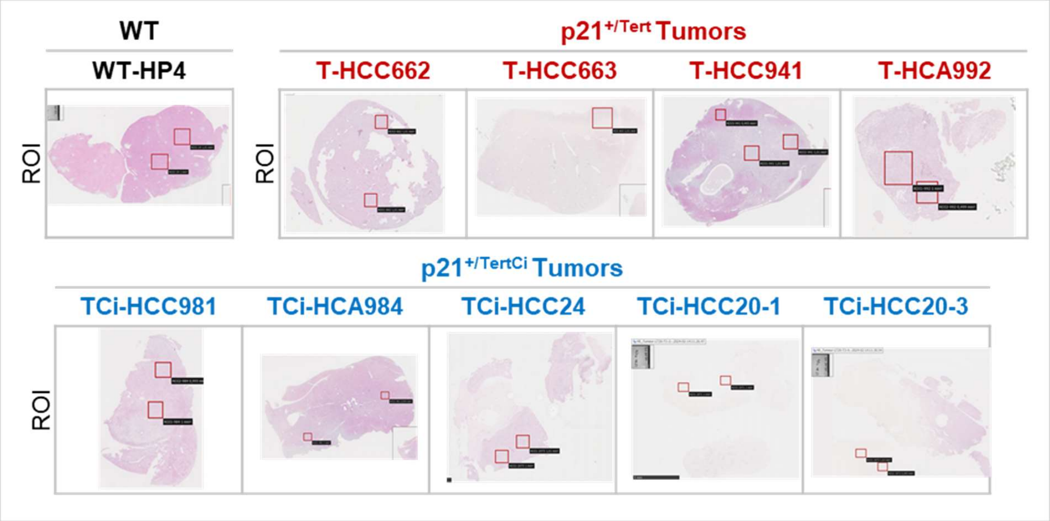

Reviewer #2

Summary: Braud et al. explore the implication of non-canonical signaling pathway of TERT expression in liver carcinogenesis, they generated two mouse models under p21 promoter, one expresses an active form of TERT while the other expresses an inactive form. Both models develop liver tumors at 18-20 months old mice. The study is descriptive of those tumors. The model implies the expression of TERT in dormant cells (G0/G1) as P21 is expressed in these cells. When TERT is expressed, it can degrade p21 as it is demonstrated in the literature, leading to a proliferative state. We can therefore understand the development of tumors in the TERT-WT model, interestingly, they find tumors in the TERT-inactive model as well, which suggest an oncogenic activity of TERT beyond telomere maintenance. Nevertheless, the presence of tumors in the control group mitigates the results as we are not aware of the number of animals used for the whole study, as well as what other factors might generate tumors especially in old mice. The main statement of the article is new and valid but any further interpretation is compromised as the authors do not go further in validating the WES and RNAseq data. The authors remain with a descriptive and literature validation approach. WES, RNAseq data give indications to potential targets, for example Figure 5 suggests that glycolysis and NRF2 are differentially expressed in the tumors, we need further validation at protein or mRNA level. Even if further work is needed, the study is of interest to the field of genetics of liver cancer. It suggests a new role of TERT. The model is ingenious and interesting to explore the implication of dormant cells in cancer development.

We thank referee 2 for his/her comments. In response to Referee 2’s comments, all figures have been revised to improve their quality and readability (See below).

Major comments

1. How did you check that the TERT_Ci is really inactive? As from the WES and RNAseq, we don't see differences in TERT expression.

See our response to Referee 1, point 1.

2. Table1, the wild-type mice develop liver hyperplasia and hemangiosarcoma, how many control mice were used, how do you differentiate between the spontaneous disease and the induced malignancy due to the vector

During our studies on the effect of Tert and $Tert^{Ci}$ expression in the lungs of aged mice, we analyzed cohorts (>50 animals) of WT and $p21^{+/-}$ mice. Only, one WT mouse develop non-generative hepatocellular hyperplasia and one develop Hemangiosarcoma. No tumors were observed in $p21^{+/-}$ mice. This spectrum and frequency of liver hyperplasia and hemangiosarcoma observed in control mice are consistent with previously reported spontaneous, age-related lesions, whereas tumors arising in $p21^{+/Tert}$ and $p21^{+/TertCi}$ displayed distinct features and increased incidence.

3. Fig 4 and Fig5, the clustering of the tumors is not demonstrated, we see a partial clustering of the genes that are supposed to form signatures, but the tumors are not clustered. Another analysis of the data should be performed to determine if the different tumors cluster by group.

We thank Referee 2 for raising this concern, which we fully acknowledge. In response, we have performed tumor clustering (**new Figure 4A**) and principal component analysis (**Figure S3**). The clustering reveals that T-HCC662, T-HCC663, and TCi-HCC201 form a distinct group, indicating that TCi-HCC201, harboring the Ep300Ala74Glu mutation, shares transcriptional similarities with the two β-catenin–activated tumors.

A second cluster includes T-HCA92, T-HCC941, TCi-HCA984, TCi-HCC981, TCi-HCC24, and TCi-HCC203. Notably, T-HCA92 and T-HCC941 cluster with TCi-HCC tumors carrying the hRASGln61His mutation. Within this cluster, TCi-HCC203 harboring both Ep300Ala74Glu and hRASGln61His displays an intermediate and distinct transcriptional profile.

These results have been incorporated into the revised manuscript **Line 268**.

4. Authors state that in figure 5A, all tumors overexpress glycolytic enzymes, this cannot be deduced from the figure (group 2 genes are mostly blue = downregulated)

We thank Referee 2 for highlighting this issue and agree that the original Figure 5A was unclear. We have generated a revised Figure 5A in which glycolytic and gluconeogenic genes are explicitly distinguished. To improve clarity and interpretability, the updated analysis focuses exclusively on HCC tumors. Consistent with the original figure, all HCC tumors show downregulation of the gluconeogenic genes *AldoB* and *Fbp1*.

Regarding glycolytic genes, TCi-HCC203, T-HCC941, and TCi-HCC24 exhibit broad upregulation of a large set of glycolytic genes, whereas the remaining tumors display increased expression of a more limited subset, including *Hk2* which is consistently overexpressed across these tumors.

This revised analysis, which more accurately reflects glycolytic enzyme expression and reinforces the downregulation of *Fbp1* and *AldoB*, has been incorporated into the manuscript. Notably, *AldoB* has been reported to stably associate with *Fbp1* (Li Gu et al., *Cell Metabolism*, 2023).

These results have been incorporated into the revised manuscript **Line 322.**

5&6. Here the authors state that LT662 and LT663, Fig5C, express group 1 genes, we cannot view the group on the graph, therefore we cannot come to the same conclusion. The conclusions on Glycolysis and NRF2 role are not solid as displayed. I suggest a better presentation of the results with a clustering of the tumors to come to a signature associated with each tumor model.

In response to Referee 2's concern, we generated a **revised Figure 5C** including only HCC tumors, providing a more accurate view of *Nrf2* target gene expression.

Clustering analysis did not reveal a tumor model–specific signature. Instead, it identified groups of genes commonly expressed across subsets of tumors that distinguish β-catenin-activated tumors from hRAS-activated tumors. Specifically, Groups 1 and 2 are predominantly expressed in T-HCC662 and T-

HCC663, whereas Group 3 genes are mainly observed in T-HCC663 and TCi-HCC201 somehow consistent with tumor clustering shown in Figure 4A. Group 4 genes are primarily overexpressed in TCi-HCC203, T-HCC941, and TCi-HCC981, as well as in T-HCC663, TCi-HCC24, and TCi-HCC203.

The expression profiles point to a coordinated ferroptosis-protective program driven by distinct sets of genes. In Figure 5C, genes associated with increased resistance to ferroptotic cell death are highlighted: those mainly overexpressed in Groups 1, 2, and 3 are shown in red (HmoX1, Fth1, Taldo1, Tm4sf20, Akr1b10, Tkt, Rrm2, Nupr1, while those preferentially overexpressed in Groups 4, 5, and 6 are shown in blue (*Slc7a11, Gpx2, Gsr, Nqo1, Cbr3, Gsta1, Cul3 Sqtsm1, Gpx2, Gsr)*. Although there is some redundancy between the tumors, these results suggest that beta-catenin tumors and TCi-HCC201 resists ferroptosis primarily by limiting free iron and boost NADPH production, whereas MAPK activated tumors rely on a canonical NRF2-SLC7A11-glutathione axis to detoxify lipid peroxides through enhanced antioxidant enzyme activity.

These results have been incorporated into the revised manuscript **Line 333**.

7. As the model relies on p21 expression, did you check for senescence?

We assessed p21 expression using RNA sequencing and immunostaining only.

Minor comments

8. Line 157, there is an error displayed

We identified the error, which was due to a broken Word link that did not function properly. This issue has now been corrected in the revised version of the manuscript.

9. Line 245, "et" written is French

We corrected this mistake.

10. Line 246 to Line 253, wrong referencing of the panels in the figure 3. 3C should be 3D and 3D should be 3E

We corrected this wrong referencing **Line 257** and **Line 259**.

11. Respect the nomenclature for gene names and protein names should be observed.

We respect gene nomenclature in the revised version.

12. The categories of tumors (WT, TERT or TERT-Ci) should be recalled in the figures to facilitate their interpretation, in addition of the acronym HCC, HCA or HP for each tumor. For example, LT662_HCC or HCC662.

Thank you for this suggestion. To improve clarity and facilitate figure interpretation, we have revised the nomenclature used in the figures. Tumors are now labeled according to their category, using “T” or “TCi” to indicate TERT or $TERT^{Ci}$ or WT genotype, followed by the tumor type (HCC, HCA or HP), and the mouse number (e.g., TCi-HCC982).

This updated naming convention makes the classification of each sample more explicit and has been applied consistently across all figures and throughout the manuscript.

13. To decrease the number of figures, the microscopy images can be reduced to one representative image per type of tumor or category of interest. The rest should appear in the supplementary.

Reducing the microscopy images to a single representative per tumor type is challenging, as there is notable heterogeneity among tumors within the same category ($p21^{+/Tert}$ and $p21^{+/TertCi}$).

14. Figure 8 and 9 should be merged together.

Figure 8 and 9 have been merged accordingly.

15. Table S1 does not display all tumors or tissues describe in the paper (L1069-2, L982-2), in relation to this, try to show always the same tumors in all figures, there are some where we only see a fraction without justification.

L1069-2 and L982-2 are normal liver samples from $p21^{+/Tert}$ and $p21^{+/TertCi}$ mice, respectively. They were used as controls to assess tumor architecture using the Hyperion™ Imaging System. Table S1 lists additional $p21^{+/Tert}$ and $p21^{+/TertCi}$ samples corresponding to non-tumoral liver regions.

16. The discussion is lengthy and should highlight only important points.

The discussion was reduced by 20% accordingly to highlight the most important points.

Reviewer #3 (Comments to the Authors (Required)):

The authors of this article have used two mice models previously described that express the TERT gene coding region under control of the p21 promoter. One of the models express the wild-type TERT protein while the other express one catalytically inactive variant of this protein. The authors report that mice from both models develop liver tumors by 18-20 months of age. They conclude from this observation that TERT expression drives liver tumorigenesis independently of the catalytic activity of the enzyme and, therefore, of their telomere functions. In addition, the authors characterize extensively the liver tumors generated showing that they present histopathological features resembling human hepatocellular carcinomas (HCCs). The presence of driver mutations in these tumors, their transcriptome profile, signaling pathways activation and immune cells infiltration are also carefully analyzed. One interesting observation is that different driver mutations are found in the two mice models. Activating mutations in the Ctnnb1 gene, coding for β-catenin and in the genes coding for PP1 (protein phosphatase 1) subunits were observed in the tumors over-expressing wild type TERT. In contrast, the activating Hras mutation (HrasGln61Lys) was observed in several tumors over-expressing the mutant TertCi protein. These tumors also carried mutations in PP1 subunits and three of them the Ep300Ala74Glu variant. Tumors from both mice models showed increased glycolysis, suppression of gluconeogenesis, reduced HNF-4α-positive hepatocytes and diminished immune cells infiltration. The authors conclude that "these findings uncover telomere-independent functions of TERT and identify molecular and metabolic features with potential relevance for predicting immunotherapy response". The data reported in this article are extensive and very sound. Even so, there are some questions that still remain open about the role of TERT and the inactive mutant variant, on liver tumorigenesis. Current models propose that tumor development require some mechanism of telomere elongation being induced. Many tumors activate TERT expression to induce telomere elongation. This mechanism could be mimicked upon TERT expression under the control of p21 promoter in senescent cells, as discussed by the authors. In the case of the expression of the TERT mutant this mechanism of direct telomere elongation is discarded.
We thank referee 3 for his/her comments.

Major points

(1) One possible mechanism of elongation could be over-expression of the endogenous TERT gene but the data presented in the article rather indicated a decrease in TERT expression. It would be very interesting to determine the size of the telomeres in the tumors present in the mice that overexpressed the TERT mutant and to compare it with that of the tumors induced by wild-type TERT. In the case that telomere elongation is observed after mutant TERT expression is would be very interesting to consider the mechanisms involved given that the expression of endogenous TERT is not induced. Could the alternative mechanism of telomere elongation (ALT) be induced? Telomere length analysis in the tumors in the two mice models could provide some information about these possible alternatives.

This is an important question that we also considered. In our previous study (De Nonneville et al., EMBO Molecular Medicine (2022)), we showed that ALT osteosarcoma tumors display distinct profiles in TeSLA assays, characterized by a clear increase in upper bands. This pattern was not observed in the few TeSLA experiments we performed in the present study (see our response to Reviewer 1).

$p21^{+/TertCi}$ tumors display shorter telomeres than $p21^{+/TertCi}$ tumors. In addition, in human cancers, ALT is observed predominantly in tumors of mesenchymal origin. Its prevalence in carcinomas is very low, although we do not exclude that it may occur in specific contexts.

(2) A second mechanistic question still open is the reason by overexpression of TERT or the mutant protein induce different tumorigenic pathways. Could these two protein variants interact with different molecular targets? Could be these differences due to the relatively small number of tumors analyzed? These and other possible alternatives could be considered in more detail in the Discussion section.

We fully agree with Referee 3. The relatively small number of tumors analyzed represents a limitation of this study and may contribute to the observation that overexpression of TERT or $TERT^{Ci}$ appears to engage distinct tumorigenic pathways. We now acknowledge this point in the Discussion **Line 466**. Nevertheless, it is notable that 2 of 4 $p21^{+/Tert}$ tumors and 3 of 5 $p21^{+/TertCi}$ tumors exhibit activation of β-catenin and HRAS pathways, respectively.

Minor points

1. **The proportion of mice that develop liver tumors is estimated in 25% in the Abstract section (line 34) and 15% in the Introduction (line 88)**

Thank you for pointing out this inconsistency. It has now been corrected in the Abstract to ensure consistency with the value reported in the Introduction.

2. **There is a typographic mistake in line 157**

We identified the error, which was due to a broken Word link that did not function properly. This issue has now been corrected in the revised version of the manuscript.

3. **Line 206, "reported approx." might be "reported in approx."**

We corrected this mistake **Line 212**.

4. **There is a typographic mistake in line 245 "35% et 45%)**

We corrected this mistake **Line 253**.

5. **Line 663. The statement "was not blinded" should be revised.**

This statement was corrected **Line 627**.

June 1, 2026

RE: Life Science Alliance Manuscript #LSA-2026-03660-TR

Dr. Vincent Géli
CNRS
CRCM
27 bd Leï roure
marseille 13009
France

Dear Dr. Géli,

Thank you for submitting your revised manuscript entitled "TERT drives liver tumorigenesis beyond telomere elongation". I appreciate your patience in waiting to hear our decision, due in part to my recent travels. As you will see, all reviewers are entirely satisfied. We would be happy to publish your paper in Life Science Alliance pending final revisions necessary to meet our formatting guidelines detailed below. We also invite you to consider the minor suggestions by Reviewer 3.

MANUSCRIPT ORGANIZATION AND FORMATTING:

To avoid unnecessary delays in the acceptance and publication of your paper, please read the following information carefully. Full guidelines are available on our Instructions for Authors page, https://www.life-science-alliance.org/authors

Submission of a paper that does not conform to Life Science Alliance guidelines will delay the acceptance of your manuscript.

-We appreciate the brevity of the current title, but we advocate refining it to provide greater specificity than is currently conveyed by the word "beyond". We suggest you consider "TERT drives liver tumorigenesis independent of telomere elongation" if you feel this wording is accurate. This change is left entirely to your discretion.
-Please add the X and Bluesky handles of your host institute/organization, as well as your own, and/or one of the authors, in our system.
-Please be sure that the authorship listing and order are correct and match between the system and the manuscript file.
-Please rename "Disclosure and competing interest statement" to "Conflict of Interest."
-Please add your main, supplementary figure, and table legends to the main manuscript text after the references section.
-There are call-outs for figures 6A and B in the manuscript text. However, this figure doesn't have labeled panels, nor are they mentioned in its legend. Please correct.
-We encourage you to revise the figure legends for Figure S1 such that the figure panels are introduced in alphabetical order.
-Please add a callout for Figure 4H to your main manuscript text.
-Please add scale bar(s) to the images in Figure 6.
-Please update the Data Availability section with accession numbers for RNA seq data.
-Please upload Tables S1-S4 as Table files rather than as "related manuscript files". Kindly contact the journal office with any questions here.
-Image integrity screening identified potential reuse of samples for the images shown in Figure 7 and Figure 8. This reuse is acceptable, but should be noted in the legends for both figures.

We encourage our authors to provide original source data, particularly uncropped/-processed electrophoretic blots and spreadsheets for the main figures of the manuscript. If you would like to add source data, we would welcome one PDF/Excel-file per figure for this information. These files will be linked as supplementary "Source Data" files.

It is Life Science Alliance policy that if requested, original data images must be made available to the editors. Failure to provide original images upon request will result in unavoidable delays in publication. Please ensure that you have access to all original data images prior to final submission.

If you are planning a press release on your work, please inform us immediately to allow informing our production team and scheduling a release date.

We welcome submissions of potential cover images for the issue of LSA in which your work would appear. If you have high quality images associated with this work, please feel free to email these, with a caption, to the journal office.

LSA encourages authors to provide a 30-60 second video where the study is briefly explained. These videos will be appear embedded with the manuscript online at Life Science Alliance, and on social media to promote the published paper and authors

(for examples, see https://docs.google.com/document/d/1-UWCfbE4pGcDdcgzcmiuJl2XMBJnxKYeqRvLLrLSo8s/edit?usp=sharing). Corresponding or first-authors are welcome to submit the video. Please submit only one video per manuscript. The video can be emailed to contact@life-science-alliance.org

FINAL FILES:

To upload the final version of your manuscript, please log in to your account: https://lsa.msubmit.net/cgi-bin/main.plex
You will be guided to complete the submission of your revised manuscript and to fill in all necessary information. Please get in touch in case you do not know or remember your login name.

The following items are required for acceptance.

-- An editable version of the final text (.DOC or .DOCX) is needed for copyediting (no PDFs).

-- High-resolution figure, supplementary figure and video files uploaded as individual files: See our detailed guidelines for preparing your production-ready images, https://www.life-science-alliance.org/authors

-- Summary blurb (enter in submission system): A short text summarizing in a single sentence the study (max. 200 characters including spaces). This text is used in conjunction with the titles of papers, hence should be informative and complementary to the title. It should describe the context and significance of the findings for a general readership; it should be written in the present tense and refer to the work in the third person. Author names should not be mentioned.

The license to publish form must be signed before your manuscript can be sent to production. A link to the license to publish form will be available to the corresponding author only. Please take a moment to check your funder requirements.

Reviews, decision letters, and point-by-point responses associated with peer-review at Life Science Alliance will be published, alongside the manuscript. If you do want to opt out of having the reviewer reports and your point-by-point responses displayed, please let us know immediately.

Thank you for your attention to these final processing requirements. Please revise and format the manuscript and upload materials as soon as you are able.

Thank you for this interesting contribution to the literature. We look forward to publishing your paper in Life Science Alliance.

Sincerely,

Tim Fessenden
Scientific Editor
Life Science Alliance

Reviewer #1 (Comments to the Authors (Required)):

the authors have done wonderful job in revising the paper. I think it is good to be published now.

Reviewer #2 (Comments to the Authors (Required)):

The authors have answered all my conerns, the manuscript is now very clear and will be of interest to the community. I thank the authors for their consideration.

Reviewer #3 (Comments to the Authors (Required)):

The authors of the manuscript have addressed all my previous concerns and comments and I have no additional ones.

I have noticed a few minor points, as follows:

- The sentence in line 574 "Of note, p21 remained elevated in the tumours" is repeated in the following paragraph.
- The link shown in lines 679-680 seems to be also repeated twice
- Line 855, "CD4" is repeated twice and in one of them the authors might refer to "CD8".
- It would be helpful if panels C and D of Figure S1 would show the same lines drawn in panels A and B to indicated the samples

and fragments smaller than 1 kb.

June 11, 2026

RE: Life Science Alliance Manuscript #LSA-2026-03660-TRR

Dr. Vincent Géli
Centre National de la Recherche Scientifique
CRCM
27 Bd Lei Roure
Marseille 13273
France

Dear Dr. Géli,

Thank you for submitting your Research Article entitled "TERT drives liver tumorigenesis beyond telomere elongation". It is a pleasure to let you know that your manuscript is now accepted for publication in Life Science Alliance. Congratulations on this interesting work. I personally appreciate that you felt LSA would be an appropriate venue and I enjoyed working with you to publish these important findings.

The final published version of your manuscript will be deposited by us to PubMed Central upon publication.

Your manuscript will now progress through copyediting and proofing. It is journal policy that authors provide original data upon request.

Reviews, decision letters, and point-by-point responses associated with peer-review at Life Science Alliance will be published, alongside the manuscript. If you do want to opt out of having the reviewer reports and your point-by-point responses displayed, please let us know immediately.

IMPORTANT: If you will be unreachable at any time, please provide us with the email address of an alternate author. Failure to respond to routine queries may lead to unavoidable delays in publication.

Scheduling details will be available from our production department. You will receive proofs shortly before the publication date. Only essential corrections can be made at the proof stage so if there are any minor final changes you wish to make to the manuscript, please let the journal office know now.

DISTRIBUTION OF MATERIALS:
Authors are required to distribute freely any materials used in experiments published in Life Science Alliance. Authors are encouraged to deposit materials used in their studies to the appropriate repositories for distribution to researchers.

You can contact the journal office with any questions, contact@life-science-alliance.org

Again, congratulations on a very nice paper. I hope you found the review process to be constructive and are pleased with how the manuscript was handled editorially. We look forward to future exciting submissions from your lab.

Sincerely,

Tim Fessenden
Scientific Editor
Life Science Alliance
